# Engineered red blood cells as an off-the-shelf allogeneic anti-tumor therapeutic

**DOI:** 10.1038/s41467-021-22898-3

**Published:** 2021-05-11

**Authors:** Xuqing Zhang, Mengyao Luo, Shamael R. Dastagir, Mellissa Nixon, Annie Khamhoung, Andrea Schmidt, Albert Lee, Naren Subbiah, Douglas C. McLaughlin, Christopher L. Moore, Mary Gribble, Nicholas Bayhi, Viral Amin, Ryan Pepi, Sneha Pawar, Timothy J. Lyford, Vikram Soman, Jennifer Mellen, Christopher L. Carpenter, Laurence A. Turka, Thomas J. Wickham, Tiffany F. Chen

**Affiliations:** Rubius Therapeutics, Inc., Cambridge, MA USA

**Keywords:** Applied immunology, Tumour immunology, Tumour immunology

## Abstract

Checkpoint inhibitors and T-cell therapies have highlighted the critical role of T cells in anti-cancer immunity. However, limitations associated with these treatments drive the need for alternative approaches. Here, we engineer red blood cells into artificial antigen-presenting cells (aAPCs) presenting a peptide bound to the major histocompatibility complex I, the costimulatory ligand 4-1BBL, and interleukin (IL)-12. This leads to robust, antigen-specific T-cell expansion, memory formation, additional immune activation, tumor control, and antigen spreading in tumor models in vivo. The presence of 4-1BBL and IL-12 induces minimal toxicities due to restriction to the vasculature and spleen. The allogeneic aAPC, RTX-321, comprised of human leukocyte antigen-A*02:01 presenting the human papilloma virus (HPV) peptide HPV16 E7_11-19_, 4-1BBL, and IL-12 on the surface, activates HPV-specific T cells and promotes effector function in vitro. Thus, RTX-321 is a potential ‘off-the-shelf’ in vivo cellular immunotherapy for treating HPV + cancers, including cervical and head/neck cancers.

## Abstract (web summary)

Red blood cells (RBCs) have unique properties that have been exploited for therapeutic uses. Here the authors engineer RBCs to co-express tumor associated antigens on MHC I, 4-1BBL and IL-12, generating artificial antigen presenting cells that can induce antigen-specific T cell responses and antitumor immune responses in preclinical models.

## Introduction

T cells play a critical role in anticancer immunity through the specific recognition of cancer antigens by T-cell receptors (TCRs)^1^. Approaches designed to manipulate or mimic the T-cell response (such as checkpoint inhibitors and chimeric antigen receptor T [CAR-T] cells, respectively) have been an important focus of anticancer therapeutics in recent years. However, the limitations associated with these treatments (including fatal adverse effects^2–4^ and the development of resistance in some cases^5,6^), as well as the complex and costly personalized production processes required for autologous CAR-T cells^7–9^, have driven continued investigation into alternative approaches to stimulate T-cell-mediated antitumor responses

Effective T-cell activation requires three distinct signals: engagement of the TCR by a peptide bound to a major histocompatibility complex (MHC) molecule (signal 1); a costimulatory signal to promote T-cell activation, function, and survival (signal 2); and a cytokine signal to facilitate further expansion and differentiation of T cells, enhanced effector function and the formation of immunological memory (signal 3)^10,11^. Signals 1 and 2 are typically delivered by antigen-presenting cells (APCs)^12^. Dendritic cells or artificial APC (aAPC) platforms utilizing fibroblasts, microbeads, biodegradable polymers, and K562 cells have been investigated as anticancer therapeutics to stimulate T cells either in vivo or ex vivo^13–16^.

Red blood cells (RBCs) have unique properties that make them attractive for allogeneic cell therapy, including inherent biocompatibility when O-negative donor blood is utilized^17^. This has led to their long-term use and familiar history in transfusion medicine with very few side effects^18^. Moreover, RBCs are associated with multiple mechanisms of immune privilege that protect them from inhibitory or adverse host responses^18^. From the manufacturing standpoint, cell culture and differentiation of hematopoietic progenitor cells allows for many-fold expansion of erythroid cells. In addition, via genetic modification^19^, it is possible to generate engineered human RBCs that express biotherapeutic proteins, herein termed Red Cell Therapeutics^™®^ or RCTs^™^. As erythroid precursor cells enucleate, the expressed protein is maintained, but the genetic modifications performed on progenitor cells are not carried into the final product intended for patient administration^19^. Lastly, since RBCs are confined to the vasculature in most parenchymal organs and the spleen, they have the potential to avoid some of the on-target toxicities seen with circulating immunomodulatory proteins^20,21^.

Here, we describe the development, characterization, and preclinical testing of our allogeneic, Red Cell Therapeutics aAPC platform (RCT-aAPC) comprised of a tumor-specific peptide bound to MHC class I, a costimulatory ligand (4-1BBL) and a cytokine signal, interleukin 12 (IL-12). We demonstrate the ability of such an RCT-aAPC to drive antigen-specific T-cell expansion and acquisition of effector function both in vitro and in vivo, and to control tumors in preclinical mouse models. Importantly, tumor control is associated with the development of long-term memory and epitope spreading, leading to efficacy against tumors that do not express the original target antigen but are otherwise identical. These functions led to the creation of a clinical candidate, RTX^™^-321, which expresses human leukocyte antigen (HLA)-A*02:01 with human papillomavirus (HPV) 16 E7 peptide _11–19_ (HLA-A2-HPV), 4-1BBL, and IL-12. We show that RTX-321 induces the activation of HPV antigen-specific primary human T cells, and that all three signals are sufficient for robust effector function and differentiation of effector memory cells. Given the poor survival rates associated with some recurrent HPV^+^ cancers^22^, our study shows that RTX-321 represents a promising strategy for clinical investigation in multiple tumor types.

## Results

### RCT-aAPCs generate functional CD8^+^ T cells in vitro

**Fig. 1:**
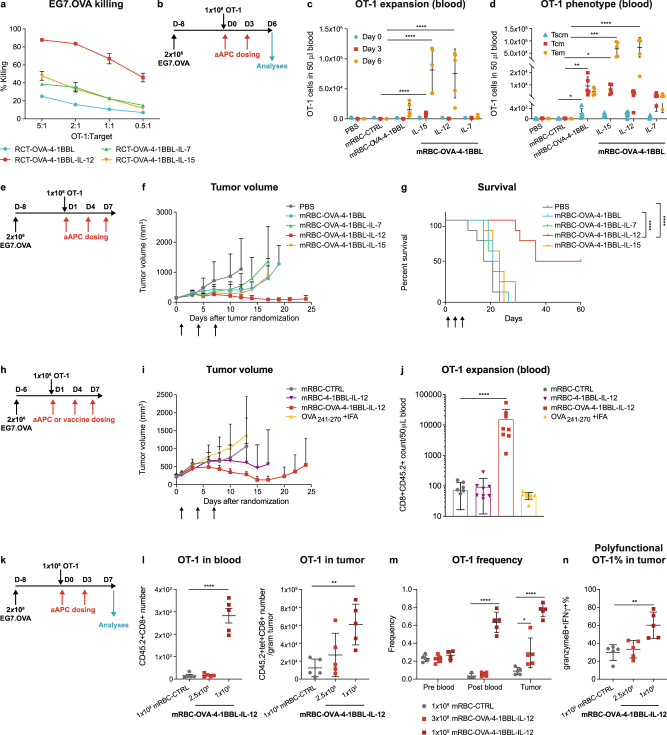
RCT-aAPCs and mRBC-aAPCs promote antigen-specific T-cell expansion, memory formation, and cytotoxicity towards tumor cells in vitro and in vivo. **a** EG7.OVA percent killing by RCT-activated OT-1 cells (*n* = 3). **b** CD45.1 Pep Boy mice were randomized (~180 mm^3^; *n* = 5), treated with naïve OT-1 cells, and dosed with 1 × 10^9^ mRBC-CTRL or mRBC-aAPC. **c** OT-1 number on days 0, 3, and 6. One-way ANOVA compared to mRBC-CTRL; Day 3: mRBC-OVA-4-1BBL-IL-15 *P* < 0.0001; Day 6: mRBC-OVA-4-1BBL-IL-15 *P* < 0.0001, mRBC-OVA-4-1BBL-IL-12 *P* = 0.0001. **d** Memory OT-1 number on day 6 in blood. One-way ANOVA compared to mRBC-CTRL; T_scm_ (stem memory T cells): mRBC-OVA-4-1BBL *P* = 0.038; T_cm_: mRBC-OVA-4-1BBL *P* = 0.0016, mRBC-OVA-4-1BBL-IL-15 *P* = 0.025; T_em_: mRBC-OVA-4-1BBL-IL-15 *P* = 0.0001, mRBC-OVA-4-1BBL-IL-15 *P* < 0.0001. **e** CD45.1 Pep Boy mice were randomized (~150 mm^3^; *n* = 8), treated with naïve OT-1 cells, and dosed with 2.5 × 10^8^ mRBC-CTRL or mRBC-aAPC. **f** Tumor growth and **g** survival. Log-rank (Mantel-Cox, one-sided) test of survival curve; mRBC-OVA-4-1BBL vs mRBC-OVA-4-1BBL-IL-12 and PBS vs mRBC-OVA-4-1BBL-IL-12 *P* < 0.0001. **h** CD45.1 Pep Boy mice were randomized (~230 mm^3^; *n* = 8), treated with naïve OT-1 cells, and dosed with OVA_241-270_ peptide with IFA or 1 × 10^9^ mRBC-CTRL, mRBC-4-1BBL-IL-12, or mRBC-OVA-4-1BBL-IL-12. **i** Tumor growth. **j** OT-1 number in blood on Day 6. One-way ANOVA compared to mRBC-CTRL; mRBC-OVA-4-1BBL-IL-12 *P* < 0.0001. **k** CD45.1 Pep Boy mice were randomized (~175 mm^3^; *n* = 5), treated with naïve OT-1 cells, and dosed with 1 × 10^9^ mRBC-CTRL or a dose titration of mRBC-OVA-4-1BBL-IL-12 (1 × 10^9^, 2.5 × 10^8^). **l** OT-1 numbers in 50 μL blood and per gram of tumor on day 7. One-way ANOVA compared to mRBC-CTRL; blood: 1 × 10^9^ *P* < 0.0001; tumor: 1 × 10^9^ *P* = 0.0046. **m** TCRβ sequencing analyses of OT-1 TCR frequency in blood on day 0 (pre-blood), on day 7 (post-blood), and in tumor on day 7. One-way ANOVA compared to mRBC-CTRL; post blood: 1 × 10^9^ *P* < 0.0001; tumor: 3 × 10^8^ *P* = 0.02, 1 × 10^9^ *P* < 0.0001. **n** Polyfunctionality (granzyme B^+^IFNγ^+^ %) in the tumor-infiltrating OT-1 cells on day 7. One-way ANOVA compared to mRBC-CTRL; 1 × 10^9^ *P* = 0.0021. Data are depicted as mean ± s.d. and are representative of four (**l** blood), two (**a**, **c**, **d**, **f**, **g**, **l** tumor, **n**), or one (**i**, **j**, **m**) independent experiments. Source data are provided as a Source Data file.

Human CD34^+^ hematopoietic progenitor cells were expanded and transduced with lentivirus, followed by expansion and differentiation into enucleated engineered RBCs, to generate RCT-aAPCs (Supplementary Fig. 1a). We produced RCT-aAPCs using lentiviral vectors encoding a single chain trimer of the murine MHC class I molecule H-2K^b^, and beta-2 microglobulin (β2M) fused with an ovalbumin-specific peptide SIINFEKL (OVA) (signal 1), 4-1BBL (signal 2), and cytokines IL-7 (RCT-OVA-4-1BBL-IL-7), IL-12 (RCT-OVA-4-1BBL-IL-12), or IL-15 (RCT-OVA-4-1BBL-IL-15) as signal 3. To assess the effects of various RCT-aAPCs on antigen-specific T cells, we used OT-1 cells, a TCR transgenic population expressing a receptor that recognizes OVA peptide bound to H-2K^b^. OT-1 cells treated with RCT-aAPCs expressing all three signals had increased cytotoxicity towards OVA-expressing EG7.OVA tumor cells compared with those treated with RCT-OVA-4-1BBL, with IL-12 being the most effective cytokine tested (Fig. 1a). Minimal off-target killing occurred when parental OVA-negative EL4 cells were used as targets and signal 1 (OVA) specificity was demonstrated by lack of interaction with the irrelevant TCR of gp100-specific pmel-1 T cells (Supplementary Fig. 2).

### Effects of RCT-aAPCs on T-cell phenotype, effector function, and anti-tumor responses in vivo

Due to the limitations of evaluating RCT-aAPCs using in vivo preclinical models, including rapid clearance of human RBCs in non-human species^23^, a murine surrogate was developed by conjugating recombinant proteins to mouse RBCs (mRBCs) using click chemistry^24^ (mRBC-aAPC, Supplementary Fig. 1b). We found that mRBC-aAPCs were a suitable surrogate for genetically modified engineered RBCs, as demonstrated by similar OT-1 cell proliferation and interferon (IFN)γ production in vitro (Supplementary Fig. 1c, d). mRBC-OVA-4-1BBL-IL-12 and mRBC-OVA-4-1BBL-IL-15 induced significant expansion of circulating OT-1 cells compared with mRBC-CTRL (unmodified mouse RBCs), mRBC-OVA-4-1BBL, or mRBC-OVA-4-1BBL-IL-7 in EG7.OVA tumor-bearing mice that were administered two doses of mRBC-aAPC (Fig. 1b, c). Importantly, the OT-1 populations expanded by mRBC-OVA-4-1BBL-IL-12 and mRBC-OVA-4-1BBL-IL-15 had a predominant effector memory (T_EM_) phenotype while still maintaining a substantial central memory (T_CM_) cell population (Fig. 1d).

We next evaluated the ability of mRBC-aAPCs to control tumor growth in vivo with OT-1 transfer (Fig. 1e). At a dose of 2.5 × 10^8^ cells, mRBC-OVA-4-1BBL-mediated transient tumor growth inhibition (Fig. 1f) without extending survival (Fig. 1g) compared with phosphate-buffered saline (PBS) treated mice. The addition of IL-12, but not IL-7 or IL-15, to the cells improved tumor growth inhibition (Fig. 1f) and extended survival (Fig. 1g) compared with mRBC-OVA-4-1BBL and the PBS control, resulting in 4/8 cures. Moreover, mRBC-OVA-4-1BBL-IL-12 treatment of 2.5 × 10^8^ cells promoted increased tumor regression (6/8 mice, Supplementary Fig. 3a) at a four-fold lower dose compared with mRBC-OVA-4-1BBL treatment of 1 × 10^9^ cells (1/8 mice, Supplementary Fig. 3b, c). Targeting the tumor antigen was important for efficacy as treatment with mRBC-4-1BBL-IL-12, lacking signal 1, led to only 1/8 cures compared to treatment with mRBC-OVA-4-1BBL-IL-12, which cured all mice treated in a separate study (Supplementary Fig. 3d, e). Based on its superior efficacy over the other cytokines and its increased potency in driving tumor control at lower doses, IL-12 was selected as the optimal signal 3 for subsequent studies.

We found that treatment with mRBC-OVA-4-1BBL-IL-12 in the EG7.OVA plus OT-1 transfer model was superior to vaccination with long OVA peptide plus incomplete Freund’s adjuvant (IFA; Fig. 1i). Median overall survival (OS) following vaccination with long OVA peptide plus IFA was 17.5 days, which was comparable to the median OS observed with mRBC-CTRL (20 days). In contrast, the median OS following treatment with mRBC-OVA-4-1BBL-IL-12 was 49 days (*P* = 0.0002 vs OVA vaccine; *P* = 0.0226 vs mRBC-CTRL). Signal 1 was required for optimal efficacy as treatment with mRBC-4-1BBL-IL-12 led to a median OS of 28.5 days, which was not statistically different from mRBC-CTRL. Only mRBC-OVA-4-1BBL-IL-12 treatment led to expansion of OT-1 cells in the blood (Fig. 1j), which correlated with efficacy.

To understand the cellular mechanisms underlying mRBC-OVA-4-1BBL-IL-12 efficacy, we evaluated both OT-1 cells and endogenous immune populations in mice (Fig. 1k). We found that OT-1 cells accumulated in the circulation and increased in a dose-dependent manner in tumors following treatment with mRBC-OVA-4-1BBL-IL-12 (Fig. 1l). Consistent with this, TCRβ sequencing demonstrated that OT-1 was the most abundant T-cell clone in both blood and tumors (Fig. 1m), and treatment with mRBC-OVA-4-1BBL-IL-12 increased the percentage of polyfunctional (granzyme B^+^IFNγ^+^) tumor-infiltrating OT-1 cells (Fig. 1n). mRBC-OVA-4-1BBL-IL-12 also promoted general anti-tumor immune effects as demonstrated by increased Ki67, tumor necrosis factor (TNF)α, and IL-2 expression by endogenous CD8^+^ T cells (Supplementary Fig. 4a), increased Ki67 and granzyme B in natural killer (NK) cells in the tumor (Supplementary Fig. 4b), decreased Treg % and increased proliferating Th1 % (IFNγ + Ki67 + %) in CD4^+^ T cells (Supplementary Fig. 4c), and increased M1 macrophage % in the tumor (Supplementary Fig. 4d).

### Biodistribution and tolerability of mRBC-OVA-4-1BBL-IL-12

**Fig. 2:**
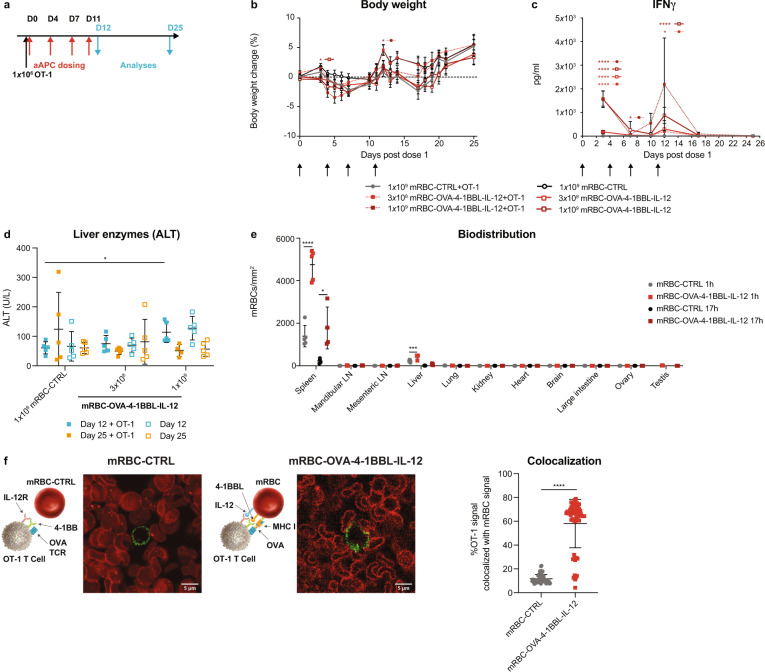
mRBC-OVA-4-1BBL-IL-12 was well tolerated, with preferential biodistribution to the spleen to facilitate T-cell interaction. **a** CD45.1 Pep Boy mice (*n* = 10) were treated with 1 × 10^6^ naïve OT-1 cells or left untreated, and either dosed with 1 × 10^9^ mRBC-CTRL or a dose titration of mRBC-OVA-4-1BBL-IL-12 (1 × 10^9^, 3 × 10^8^) on days 0, 4, 7, and 11. *n* = 5 were killed on day 12, with *n* = 5 measured for time points post day 12 for all groups except for *n* = 4 for mRBC-OVA-4-1BBL-IL-12+OT-1. **b** Body weight changes compared to day 0. Two-way ANOVA compared to mRBC-CTRL; day 3: 1 × 10^9^ *P* = 0.019; day 12: 1 × 10^9^ + OT-1 *P* = 0.048. **c** Plasma IFNγ levels over time. *n* = 10 for days 3 and 7; *n* = 5 post day 10 except for *n* = 4 for mRBC-OVA-4-1BBL-IL-12+OT-1 on days 17 and 25. Two-way ANOVA compared to mRBC-CTRL; day 3: 3 × 10^8^ and 1 × 10^9^ *P* < 0.0001, 3 × 10^8^ + OT-1 *P* = 0.0002, 1 × 10^9^ + OT-1 *P* < 0.0001; day 7: 3 × 10^8^ + OT-1 *P* = 0.018; day 12: 3 × 10^8^ + OT-1 *P* = 0.025, 1 × 10^9^ *P* < 0.0001. **d** Serum ALT levels on days 12 and 25. One-way ANOVA compared to mRBC-CTRL; day 12: 1 × 10^9^ + OT-1 *P* = 0.023. **e** CD45.1 Pep Boy mice (*n* = 5) were transferred with 2 × 10^6^ naïve CellTrace Yellow dye-labeled OT-1 cells before dosing with 1 × 10^9^ CellTrace Far Red dye-labeled mRBC-CTRL or mRBC-OVA-4-1BBL-IL-12. Fluorescently-labeled mRBCs per tissue area by immunofluorescent analyses at 1 h or 17 h post mRBC injection. One-way ANOVA compared to mRBC-CTRL; spleen: 1 h *P* < 0.0001, 17 h *P* = 0.022; liver: 1 h *P* = 0.0006. **f** Representative frame of confocal live cell imaging analyses of activated OT-1 cells (green) landing onto a layer of immobilized CellTrace Far Red dye-labeled mRBC-CTRL cells or mRBC-OVA-4-1BBL-IL-12 that were conjugated with DL650-labeled 4-1BBL protein (red). Mander’s colocalization coefficient was used to quantify percent of OT-1 signal which overlaps with mRBC signal in each frame (mRBC-CTRL: *n* = 54 frames; mRBC-OVA-4-1BBL-IL-12: *n* = 58 frames) of the live cell imaging video. Unpaired two-sided Student’s *t*-test; *P* < 0.0001. Data are depicted as mean ± s.d. and are representative of two independent experiments. Source data are provided as a Source Data file.

As toxicities have been previously observed with 4-1BB agonists^20,25^ and recombinant IL-12 in the clinic^21,26^, we performed safety measurements after repeat doses of mRBC-OVA-4-1BBL-IL-12 in mice with or without OT-1 transfer (Fig. 2a), which showed that there were no significant changes in body weight compared to control treated mice (Fig. 2b). Plasma IFNγ levels (Fig. 2c) and serum alanine aminotransferase (ALT) levels (Fig. 2d) increased during the dosing phase, with the highest dose of 1 × 10^9^ cells, but returned to baseline after a 2-week recovery period. There were minimal observed toxicities at the lower dose of 3 × 10^8^ cells, which nonetheless was sufficient for antitumor efficacy (Fig. 1f, g). Additional safety measurements, including spleen and liver weights, liver enzyme levels, liver inflammation and macrophage infiltration, hematology changes, and plasma cytokine levels, indicated similar minimal and reversible toxicity with or without OT-1 transfer (Supplementary Fig. 5). We hypothesized that the lack of significant toxicity seen with mRBC-OVA-4-1BBL-IL-12 may be due to the restricted biodistribution of RBCs compared with antibodies and cytokines. Minimal mRBC-OVA-4-1BBL-IL-12 was found in all tissues examined, with the notable exception of the spleen (Fig. 2e).

The anatomy of the splenic vasculature allows RBCs to directly interact with lymphoid cells in the parenchyma of the red pulp^27^, which led us to hypothesize that mRBC-OVA-4-1BBL-IL-12 may take part in cognate interactions with T cells in this location. Indeed, analyses of the spleen within 1 day indicated that OT-1 antigen-specific T cells were more frequently in contact with mRBC-OVA-4-1BBL-IL-12 than with mRBC-CTRL, as assessed by both colocalization (Supplementary Fig. 6a, b) and quantification of doublets by flow cytometry (Supplementary Fig. 6c). This also correlated with CD44 expression, confirming the activation of OT-1 cells (Supplementary Fig. 6d). We also demonstrated direct interactions between OT-1 cells and mRBC-OVA-4-1BBL-IL-12 by in vitro live cell imaging (Fig. 2f, Supplementary movie 1, and Supplementary movie 2). Together, these results indicate the ability of mRBC-OVA-4-1BBL-IL-12 to engage in cognate interactions with antigen-specific T cells and suggest that the spleen could be one of the primary locations for this in vivo.

### Long-term memory formation and protection against tumor rechallenge

**Fig. 3:**
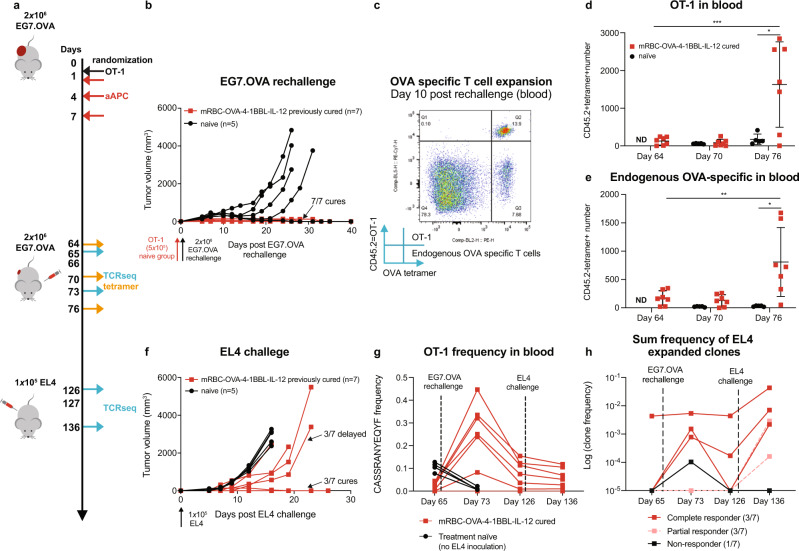
mRBC-OVA-4-1BBL-IL-12 promotes immune memory and epitope spreading, and harnesses endogenous T cells. **a** CD45.1 Pep Boy mice were randomized when EG7.OVA tumors reached ~230 mm^3^ (*n* = 8), treated with naïve OT-1 cells, and dosed with 2.5 × 10^8^ mRBC-OVA-4-1BBL-IL-12. Seven out of eight mice cured of original EG7.OVA tumors and were rechallenged on day 66 with EG7.OVA. Age-matched naïve CD45.1 Pep Boy mice (*n* = 5) were treated on day 65 with 5 × 10^5^ naïve OT-1 cells 1 day before challenge with EG7.OVA cells, as controls. **b** All previously cured mice rejected EG7.OVA rechallenge. **c** Representative flow cytometry plots showing OT-1 and endogenous OVA-specific T cells in 50 μL of peripheral blood 10 days after EG7.OVA rechallenge (Day 76). **d** OT-1 and **e** endogenous OVA-specific T-cell numbers in 50 μL peripheral blood 2 days before rechallenge (Day 64), 4 days post rechallenge (Day 70), and 10 days post rechallenge (Day 76). One-way ANOVA compared to day 64; OT-1: day 75 *P* = 0.0009; endogenous OVA-specific: day 75 *P* = 0.0079. Unpaired two-way Student’s *t* test compared to naïve; OT-1 day 75 *P* = 0.018; endogenous OVA-specific: day 75 *P* = 0.018. **f** At 61 days post-second EG7.OVA challenge on day 127, cured mice (*n* = 7) along with age-matched naïve control mice (*n* = 5) were challenged with EL4. Three out of seven cured mice had delayed EL4 growth and three out of seven rejected EL4. **g** TCRβ sequencing analyses of OT-1 frequency on days 65, 73, 126, and 136 in the blood. **h** The significantly expanded TCR clones after EL4 challenge were tracked throughout the tumor challenges. The log of the sum clone frequencies in individual mice is shown. Data are depicted as mean ± s.d. and are representative of two (**b**–**f**) or one (**g**, **h**) independent experiment. Source data are provided as a Source Data file.

To investigate the effects of mRBC-aAPCs on immune memory, mice with EG7.OVA tumors that were previously cured following treatment with mRBC-OVA-4-1BBL-IL-12 were rechallenged with EG7.OVA 66 days after initial tumor injection. Age-matched naïve mice were transferred with OT-1 cells 1 day before EG7.OVA challenge to represent residual background levels of OT-1 cells (Fig. 3a). Four out of five naïve mice succumbed to EG7.OVA challenge (Fig. 3b), and OT-1 cells did not expand in these mice (Fig. 3d). In contrast, all seven mice cured of EG7.OVA tumors by prior mRBC-OVA-4-1BBL-IL-12 treatment rejected EG7.OVA rechallenge without additional treatment (Fig. 3b). This was associated with OT-1 and endogenous OVA-specific T-cell expansion 10 days after tumor rechallenge (Fig. 3c–e) and demonstrates OVA-specific immunological memory formation following initial treatment with mRBC-OVA-4-1BBL-IL-12.

IL-12 has been shown to play a role in promoting epitope spreading^28^. To determine if protection against other tumor antigens in addition to OVA was established by mRBC-OVA-4-1BBL-IL-12 treatment, cured mice and age-matched naïve controls were challenged with the parental OVA-negative tumor cell line, EL4, 61 days after the second EG7.OVA challenge. Strikingly, previously cured mice showed either resistance to tumor growth (3/7) or delayed tumor growth (3/7) compared with control mice (Fig. 3f). This demonstrates that in the process of inducing an immune response to EG7.OVA, treatment with mRBC-OVA-4-1BBL-IL-12 led to epitope spreading to other tumor antigens.

TCRβ sequencing on T cells in peripheral blood showed that OT-1 clones increased in frequency after EG7.OVA challenge in previously cured mice (Fig. 3g). OT-1 frequency did not increase in treatment-naïve mice after tumor challenge, indicating that the tumor alone is insufficient to drive OT-1 cell expansion. We also evaluated the frequencies of unique TCRβ sequences in T-cell clones that significantly expanded post EL4 challenge (EL4-responsive TCR). Increased frequency of EL4-responsive TCRs upon each tumor challenge (EG7.OVA and EL4) was associated with complete responders (mice that rejected EL4 challenge), suggesting that T-cell-mediated protection against parental tumor antigens was generated prior to EL4 challenge (Fig. 3h). Partial responders (delayed tumor growth compared with naïve mice) had increases in EL4-responsive TCR frequencies after EL4 challenge but not during the EG7.OVA rechallenge, whereas the non-responder (tumor growth similar to naïve mice) had minimal increases in TCR frequencies upon EL4 challenge. Overall, the ability to control EL4 tumors correlated with the expansion of EL4-responsive TCR clones.

### EG7.OVA tumor control without OT-1 cell transfer is driven by 4-1BBL and IL-12

**Fig. 4:**
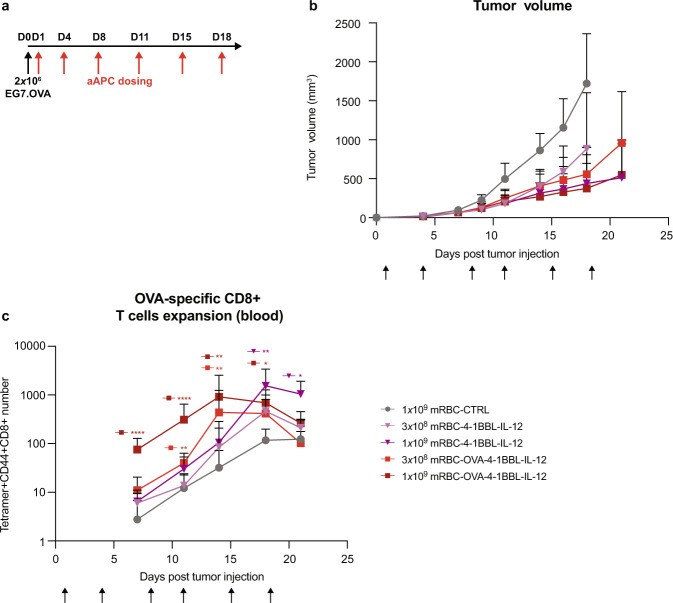
mRBC-OVA-4-1BBL-IL-12 and mRBC-4-1BBL-IL-12 delays EG7.OVA tumor growth in mice without OT-1 transfer. **a** C57BL/6 mice were inoculated subcutaneously with 2 × 10^6^ EG7.OVA cells. Following that, 1 × 10^9^ mRBC-CTRL, or a dose titration of mRBC-4-1BBL-IL-12 or mRBC-OVA-4-1BBL-IL-12 (1 × 10^9^, 3 × 10^8^) was administered (*n* = 8) on days 1, 4, 8, 11, 15, and 18. **b** Tumor growth curve after treatments. **c** OVA tetramer^+^ CD8^+^ T cells in 50 μL blood. Two-way ANOVA compared to mRBC-CTRL; 1 × 10^9^ mRBC-4-1BBL-IL-12: day 18 *P* = 0.0057, day 21 *P* = 0.014; 3 × 10^8^ mRBC-OVA-4-1BBL-IL-12: day 11 *P* = 0.0087, day 14 *P* = 0.023; 1 × 10^9^ mRBC-OVA-4-1BBL-IL-12: days 7 and 11 *P* < 0.0001, day 14 *P* = 0.006, and day 18 *P* = 0.017. Data are depicted as mean ± s.d. and are representative of two independent experiments. Source data are provided as a Source Data file.

To evaluate the ability of mRBC-OVA-4-1BBL-IL-12 to mediate tumor control in a model with minimal pre-existing OVA-specific T cells, mice were treated the day after EG7.OVA tumor injection with mRBC-OVA-4-1BBL-IL-12, mRBC-4-1BBL-IL-12, or mRBC-CTRL without OT-1 transfer (Fig. 4a). We observed that mRBC-OVA-4-1BBL-IL-12 treatment significantly delayed EG7.OVA tumor growth and increased median OS compared with mRBC-CTRL (Fig. 4b, *P* < 0.0001 and *P* = 0.0003 for the 1 × 10^9^ and 3 × 10^8^ doses, respectively). Treatment with mRBCs lacking signal 1, i.e., mRBC-4-1BBL-IL-12, also delayed tumor growth and increased median OS at both the 1 × 10^9^ and 3 × 10^8^ doses compared with mRBC-CTRL (*P* < 0.0001 and *P* = 0.013, respectively), with no statistical difference compared to mRBC-OVA-4-1BBL-IL-12. Both mRBC-OVA-4-1BBL-IL-12 and mRBC-4-1BBL-IL-12 expanded endogenous OVA-specific T cells in the blood, but the kinetics of this expansion was significantly faster when signal 1 was present, i.e., with mRBC-OVA-4-1BBL-IL-12 (Fig. 4c). Collectively, these data indicate that the presence of presented antigen on the aAPC led to a more rapid accumulation of antigen-specific T cells; however, signals 2 and 3 (4-1BBL and IL-12) were the main drivers of the anti-tumor effects observed.

### mRBC-aAPCs targeted against a tumor-associated antigen (gp100) promoted antigen-specific pmel-1 T-cell expansion and dramatically reduced B16-F10 tumor metastases

**Fig. 5:**
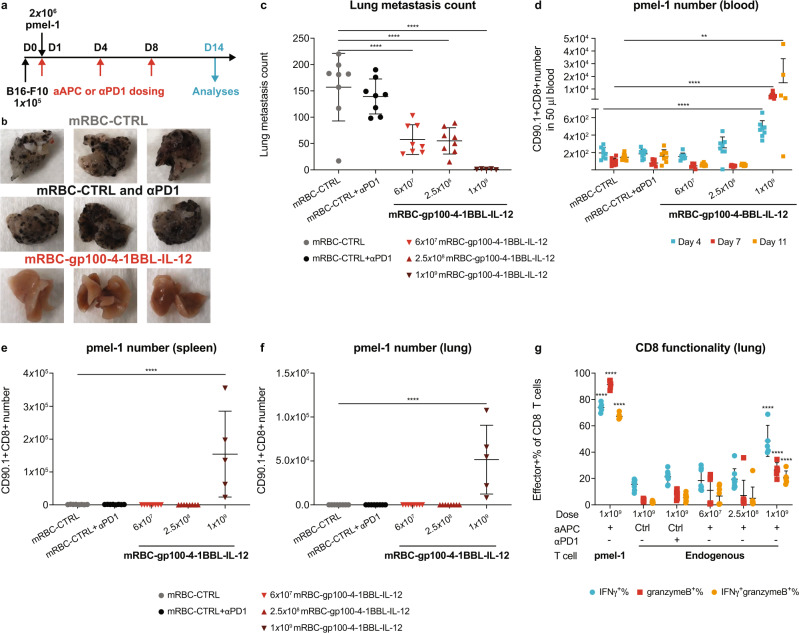
mRBC-aAPCs targeted against a tumor-associated antigen (gp100) promote antigen-specific pmel-1 T-cell expansion and effector function and dramatically reduce lung metastasis of B16-F10 tumors. **a** C57BL/6 mice were injected intravenously with 1 × 10^5^ B16-F10 tumor cells on day 0 followed by transfer of 2 × 10^6^ naïve pmel-1 T cells on day 1. Mice (*n* = 8) were then dosed with 1 × 10^9^ mRBC-CTRL, 1 × 10^9^ mRBC-CTRL with anti-PD-1 (αPD-1), or 1 × 10^9^, 2.5 × 10^8^, or 6 × 10^7^ mRBC-gp100-4-1BBL-IL-12 on days 1, 4, and 8. Analyses were performed on day 14 unless otherwise specified. For 1 × 10^9^ mRBC-gp100-4-1BBL-IL-12, *n* = 5 for day 14 analyses. **b** Representative lung photos of 1 × 10^9^ mRBC-CTRL, 1 × 10^9^ mRBC-CTRL + αPD-1, or 1 × 10^9^ mRBC-gp100-4-1BBL-IL-12-dosed mice. **c** Lung metastasis counts on day 14. One-way ANOVA *P* < 0.0001 compared to mRBC-CTRL at all dose levels. **d**–**g** pmel-1 cell number in 50 μL blood (**d**), the spleen (**e**), the left lobe of perfused lung (**f**), and the effector function of lung-infiltrating pmel-1 and endogenous CD8^+^ T cells (**g**). One-way ANOVA compared to mRBC-CTRL; blood (**d**): 1 × 10^9^ day 4 and day 7 *P* < 0.0001, day 11 *P* = 0.002; spleen (**e**) and lung (**f**): 1 × 10^9^ *P* < 0.0001; functionality (**g**): pmel-1 and endogenous CD8 at 1 × 10^9^ dose *P* < 0.0001 for IFNγ^+^%, granzymeB^+^%, and IFNγ^+^granzymeB^+^%. Data are depicted as mean ± s.d. and are representative of two independent experiments. Source data are provided as a Source Data file.

To confirm that our approach can be extended to tumor-associated antigens, mRBCs were conjugated with a different MHC I-peptide complex – H-2D^b^ loaded with the gp100 peptide (a tumor antigen expressed on B16-F10 melanoma cells), plus 4-1BBL and IL-12 to generate mRBC-gp100-4-1BBL-IL-12. In a B16-F10 lung metastasis model with pmel-1 (gp100-specific) T-cell transfer (Fig. 5a), we observed a dose-dependent effect of mRBC-gp100-4-1BBL-IL-12 treatment on lung metastases with the highest dose nearly eliminating them (Fig. 5b, c). In contrast, treatment with an anti-programmed cell death protein 1 (PD-1) antibody had no effect on lung metastases compared to mRBC-CTRL. Increased pmel-1 cells were detected in the blood, spleen, and lungs following the 1 × 10^9^ dose (Fig. 5d–f). Furthermore, treatment with mRBC-gp100-4-1BBL-IL-12 increased the total number of lung-infiltrating antigen-specific pmel-1 cells that displayed markers of effector function (IFNγ and granzyme B; Fig. 5g). Endogenous T cells also infiltrated the lungs, accompanied by an increase in IFNγ and granzyme B (Fig. 5g).

### RTX-321 treatment activates HPV-specific, TCR-transduced primary human T cells

**Fig. 6:**
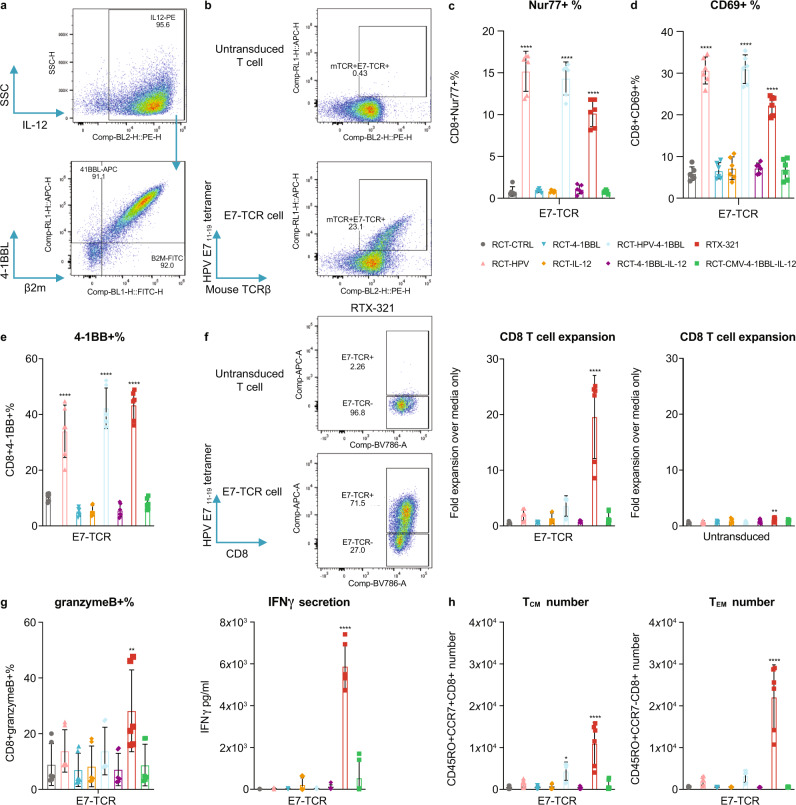
RTX-321 drives primary antigen-specific T-cell expansion, activation, memory differentiation, and effector function in vitro. **a** RTX-321 expression by anti-IL-12, anti-4-1BBL, and anti-β2M staining. **b** Representative flow plots showing TCR expression of E7-TCR cells compared with untransduced T cells before co-culture. **c**–**h** E7-TCR cells (duplicates from three donors) were incubated with RCT-CTRL, RCT-HPV, RCT-4-1BBL, RCT-IL-12, RCT-HPV-4-1BBL, RCT-4-1BBL-IL-12, RTX-321, or RCT-CMV-4-1BBL-IL-12. The Nur77^+^% at 2 h (**c**), CD69^+^% at 24 h (**d**), and 4-1BB^+^% on day 5 (**e**) of E7-TCR cells. **f** Representative flow plots showing E7-TCR^+^ expression of RCT-CTRL- and RTX-321-treated E7-TCR cells on day 5. Fold expansion of E7-TCR cells and untransduced CD8^+^ T cells over media-treated controls on day 5. **g** The granzyme B^+^ % of E7-TCR cells on day 1 and IFNγ concentration in the supernatant on day 5. **h** T_CM_ and T_EM_ of E7-TCR cell numbers on day 5. One-way ANOVA compared to RCT-CTRL; Nur77^+^% (**c**), CD69^+^% (**d**), and 4-1BB^+^% (**e**): RCT-HPV, RCT-HPV-4-1BBL, or RTX-321 *P* < 0.0001; CD8 T-cell expansion (**f**): RTX-321 E7-TCR *P* < 0.0001, untransduced *P* = 0.0062; granzymeB^+^% (**g**): RTX-321 *P* = 0.0021; IFNγ secretion (**g**): RTX-321 *P* < 0.0001; T_CM_ number (**h**): RCT-HPV-4-1BBL *P* = 0.011, RTX-321 *P* < 0.0001; T_EM_ number (**h**): RTX-321 *P* < 0.0001. Data are depicted as mean ± s.d and are representative of two independent experiments. Source data are provided as a Source Data file.

In order to treat patients with HPV16^+^ cancers, we developed RTX-321 consisting of allogeneic, cultured, human enucleated RBCs engineered to express a single chain trimer of HLA-A*02:01 and β2M fused with HPV 16 E7_11–__19_ peptide (HLA-A2-HPV; signal 1), 4-1BBL (signal 2), and IL-12 (signal 3) on the cell surface (Fig. 6a). To confirm that RTX-321 could engage and activate HPV16 E7_11–__19_-specific T cells, CD8^+^ T cells from HLA-A*02:01 donors were transduced with lentivirus to express an HPV E7-specific TCR^29^ (E7-TCR cells) (Fig. 6b) and co-cultured with RTX-321 or engineered RBCs expressing various components of RTX-321. In addition, we evaluated antigen specificity by testing an RCT-aAPC presenting a cytomegalovirus (CMV) peptide instead of the HPV 16 E7_11–__19_ peptide (RCT-CMV-4-1BBL-IL-12).

Co-culture with RTX-321 for 2 or 24 h significantly increased Nur77 and CD69 expression, respectively, on E7-TCR cells, indicating productive ligation of the TCR (Fig. 6c, d). TCR signaling was independent of 4-1BBL and IL-12 expression as engineered RBCs expressing HLA-A2-HPV alone were able to induce these activation markers (Fig. 6c, d), as well as 4-1BB (Fig. 6e). While HLA-A2-HPV alone (signal 1) was sufficient for T-cell activation, all three signals were required for T-cell expansion (Fig. 6f), which was accompanied by induction of granzyme B and IFNγ secretion (Fig. 6g). Co-culture with RTX-321, but not RCT-CMV-4-1BBL-IL-12, led to the expansion of E7-TCR cells, which demonstrates that expansion is driven by antigen specificity. The CD8^+^ T cells that expanded in response to RTX-321 consisted of both T_CM_ and T_EM_ cells (Fig. 6h). The increase in T_CM_ cells was predominantly dependent on signals 1 and 2, while all three signals contributed to the increase in T_EM_ cells. These data demonstrate that RTX-321 promotes the expansion of primary CD8^+^ antigen-specific T cells, induces the production of effector molecules, and increases T-cell activation and the generation of T_EM_ and T_CM_ phenotypes in an antigen-specific manner.

## Discussion

Here, we show that RBCs can be engineered into aAPCs that induce a tumor-specific immune response by expanding T cells against a target antigen in vivo. Of note, RTX-321 enabled the expansion of HPV-specific primary human T cells in vitro, highlighting the potential therapeutic application of this platform. This approach is designed to mimic human immunobiology through the cellular presentation of thousands of copies of biotherapeutic proteins on the cell surface of human enucleated RBCs, thus differing from other approaches that utilize synthetic receptors (as used in CAR-T cells), non-allogeneic cell lines for ex vivo T-cell expansion, or synthetic platforms (e.g. liposomes or biodegradable polymer particles)^30–32^.

In addition to a tumor antigen-loaded MHC I (signal 1), we used 4-1BBL as the T-cell costimulatory ligand (signal 2). Engagement of 4-1BB on the T cell by 4-1BBL promotes T-cell survival and proliferation, enhances effector function, and is critical for the formation of immunological memory^33^. In clinical trials of CAR-T cells, incorporation of a 4-1BB costimulatory domain favors development of central memory T cells and longer T-cell persistence compared with the use of a CD28 domain^33^.

We determined that the inclusion of IL-12 as the cytokine (signal 3) led to optimal effects by promoting antigen-specific T-cell expansion, memory and effector function, and tumor control. This is consistent with the known function of IL-12 as a potent proinflammatory cytokine that induces IFNγ release from NK cells as well as CD4^+^ and CD8^+^ T cells. Moreover, IL-12 signaling via STAT4 is essential for Th1 differentiation and the cytotoxic activity of CD8^+^ T cells^34^.

We demonstrated T-cell expansion and activation that were associated with tumor regressions and cures in two murine tumor models. Our data show that mRBC-aAPCs can drive an antitumor response not only to the foreign antigen OVA, but also to a naturally expressed tumor antigen, gp100. mRBC-aAPC treatment provided superior survival benefits compared to vaccination with long peptide plus IFA (equivalent to Montanide™ ISA 51 in clinical trials; Fig. 1i). Long peptide plus IFA is the vaccination platform furthest along in clinical development for HPV 16+ cancers^35–37^ for a single T-cell epitope and was therefore used as a comparator as opposed to other platforms, such as DNA vaccines, protein-based vaccines, and viral/bacterial vector vaccines. Our results suggest that the mRBC-aAPC format provides additional therapeutic benefits that vaccines and adjuvants may fail to provide. In addition, treatment with mRBC-aAPCs drove improved tumor control in comparison to anti-PD-1 treatment, which has been shown previously to have only a minimal effect in the B16-F10 tumor model^38–40^. Interestingly, intravenously administered mRBC-aAPCs appeared to be confined to the circulation and open vasculature of the spleen where interactions with antigen-specific T cells occurred (Supplementary Fig. 6), and proved effective at inducing an antitumor response. Indeed, recent data suggest that the spleen is an important source of cells that respond to immunotherapy^41^.

Despite showing initial signs of efficacy, IL-12 and agonist antibodies to 4-1BB have been shown to cause liver and other toxicities in clinical trials^20,21,25,26^. This is partly due to pleiotropic effects that induce immune activation at the expense of severe on-target, off-tissue toxicities^31^, which has driven attempts to restrict therapeutic immunomodulatory proteins to the tumor microenvironment and minimize systemic exposure^20^. Our mRBC-aAPCs, comprised of membrane-bound 4-1BBL and IL-12, retained a favorable safety profile demonstrated by no significant changes in mice body weight and minimal, reversible changes in splenomegaly, liver inflammation, ALT elevations, and IFNγ levels (Fig. 2b–d and Supplementary Fig. 5). This is likely due to the biodistribution of the mRBC-aAPCs to the vasculature and spleen and the membrane-bound nature of 4-1BBL and IL-12, which limits its potential for on-target, off-tissue toxicities.

Importantly, cured mice in the EG7.OVA model were not only resistant to rechallenge with EG7.OVA tumor cells but also to challenge with the parental EL4 cell line lacking OVA, indicative of both memory formation and epitope spreading^42,43^; this has been shown to improve outcomes in patients undergoing cancer immunotherapy^42^. While epitope spreading has been demonstrated previously with adoptive transfer of pre-activated^44,45^ or genetically modified T cells^28,46^, here, we find that a RBC engineered to be an artificial APC can promote epitope spreading in vivo with naïve T-cell transfer. This is a particularly promising finding in our study given that the use of a single tumor-antigen could be considered a potential limitation of our approach. For example, recent data from studies of CD19-directed immunotherapies suggest that a proportion of patients whose disease relapses can be characterized by loss of CD19 from the tumor cell surface^47^. However, our data demonstrate that the surrogate mRBC-aAPCs not only drive the activation and expansion of antigen-specific T cells but also promote changes in the tumor microenvironment, including increases in endogenous T cells, reductions in Treg numbers, increases in proliferative Th1 cells, and increases in M1 macrophages (Supplementary Fig. 4). These changes may contribute to antigen spreading and the associated protection observed in animals rechallenged with tumor cells lacking the target antigen (Fig. 3f). In addition, broad immune activation (Supplementary Fig. 4) may contribute to improved efficacy over long peptide vaccination.

Patients with HPV+ cancers have varying frequencies of HPV antigen-specific T cells in their circulation^48–50^. We modeled the range of antigen-specific T-cell frequency in patients through the presence or absence of OT-1 adoptive transfer. With a high frequency of antigen-specific T cells (i.e., with OT-1 adoptive transfer), an mRBC-aAPC that provided all three signals was more efficacious than an mRBC-aAPC lacking signal 1 (Fig. 1i and Supplementary Fig. 3d, e). However, with a lower frequency of antigen-specific T cells (i.e., without OT-1 transfer), we observed similar efficacy with mRBC-OVA-4-1BBL-IL-12 and mRBC-4-1BBL-IL-12 (Fig. 4b). This indicates that the main drivers of the anti-tumor effects in the model without OT-1 transfer are the 4-1BBL and IL-12 components of the mRBC-aAPC. Although the provision of signal 1 led to an earlier expansion of target antigen-specific T cells, this did not translate into an additional effect on tumor growth. Thus, our data indicate that the relative importance of these signals in the clinic may be determined by the number of pre-existing target antigen-specific T cells in patients.

Despite existing treatment options, there is a high unmet need in the treatment of HPV^+^ cancers, and survival of patients with recurrent disease remains poor^22^. This led us to develop an RCT-aAPC to target an HPV-specific antigen for potential therapeutic applications (RTX-321). The viral oncoprotein E7 of HPV is known to drive the development of numerous cancers through the disruption of pRB tumor suppressor activity^51^. The E7_11-19_ peptide from HPV16 was chosen for RTX-321 because it is from an invariant region of the oncoprotein^52^, demonstrated prior cytotoxic T-lymphocyte induction, and is presented by HLA-A*02:01, the most common HLA allele among the US population^53,54^. We showed that RTX-321 induced TCR signaling, led to the expansion of HPV antigen-specific T cells, promoted effector function, induced the expansion of an effector memory cell population, and established a repertoire of T_CM_ cells, suggesting that RTX-321 treatment may lead to a sustained antitumor response^55^.

Our study presents a genetically engineered RBC-based aAPC platform for the treatment of cancer and shows the activity of this approach in primary human cells. RBCs have been used in transfusion medicine for decades and O-negative donor blood can be reliably transfused to most of the population. Allogeneic engineered RBCs could be dosed across multiple patients and produced using a scalable manufacturing process without the need for the complex, personalized production processes used in TCR-T-cell and CAR-T therapies^7–9^. The RCT-aAPC platform demonstrates broad antigen applicability that is designed to mimic the biology of T-cell–APC interactions for potential application as an immunotherapeutic in a range of cancers^19,56,57^.

## Methods

### Cell lines and mice

The murine melanoma cell line B16-F10 (CRL-6475^™^), murine lymphoma cell lines EL4 (TIB-39^™^) and EG7.OVA (CRL-2113^™^), and human leukemia cell line K562 (CCL-243^™^) were obtained from American Type Culture Collection (ATCC^®^) and cultured as recommended by ATCC. Human embryonic kidney cell line Lenti-X^™^ 293T (Cat. No 632180) was obtained from Takara Bio USA, Inc. (CA, USA) and cultured as recommended by the vendor. Human embryonic kidney cell line Expi293F^™^ (Cat. No A14527) was obtained from Thermo Fisher Scientific™ (MA, USA) and cultured in line with the vendor guidelines.

Female C57BL/6J, Pep Boy (B6.SJL-Ptprca Pepcb/BoyJ), OT-1 [C57BL/6 Tg(TcraTcrb)1100Mjb/J] and pmel-1 [B6.Cg-Thy1a/Cy Tg(TcraTcrb)] mice were sourced from The Jackson Laboratory (ME, USA). Mice were maintained under specific, pathogen-free conditions in an Association for Assessment and Accreditation of Laboratory Animal Care International (AAALAC) accredited facility. The study protocols were reviewed and approved by the Charles River Laboratories (CRL; MA, USA) Institutional Animal Care and Use Committee (IACUC) or Avastus Preclinical Services (MA, USA) IACUC. Mice were maintained at both facilities on a 12-h light/12-h dark cycle. Room temperatures were maintained within the range of 73 °F + /−2°F (CRL) or 70 °F to 75 °F (Avastus). The humidity levels were maintained between 30–70% (CRL) or 35-55% (Avastus).

### Engineered RBC production and expression determination

The gene encoding the protein of interest was cloned into the multiple cloning site of a lentivirus vector under the control of the murine stem cell virus promoter sequence. Lentivirus was produced in Lenti-X 293T cells and the titer was determined in K562 cells. Human CD34^+^ hematopoietic progenitor cells derived from normal human donors, collected under Advarra Institutional Review Board (IRB) approval and informed consent, were purchased frozen from HemaCare^®^ Inc. (CA, USA). The human CD34^+^ hematopoietic progenitor cells were initially cultured in stem cell growth medium (CellGenix^®^ Inc., NH, USA) supplemented with StemSpan™ CC100 (STEMCELL Technologies, Inc., BC, Canada). Subsequently, the cells were cultured in Iscove’s Modified Dulbecco’s Medium (IMDM, Thermo Fisher Scientific) including 10 µg/mL recombinant human insulin (Sigma-Aldrich^®^, Inc., MO, USA), 200 µg/mL holo-human transferrin (BBI Solutions, ME, USA), 1 ng/mL recombinant human IL-3 (PeproTech, Inc., NJ, USA), 100 ng/mL stem cell factor (PeproTech, Inc.), and 50 ng/mL erythropoietin (PeproTech, Inc.). The cells were then cultured in IMDM including 10 µg/mL recombinant human insulin, 200 µg/mL holo-human transferrin, 10 ng/mL stem cell factor, and 16.7 ng/mL erythropoietin. Finally, the cells were cultured in IMDM including 10 µg/mL recombinant human insulin, 1 mg/mL holo-human transferrin, and 50 ng/mL erythropoietin. Cells were cultured at 37 °C in the presence of 5% CO_2_. Erythroid precursor cells were transduced with lentiviral vectors to express genes of interest. The following antibodies were used to determine expression on the surface of engineered RBCs: anti-H-2K^b^-OVA (1:100, 25-D1.16, BioLegend^®^, CA, USA), anti-mouse 4-1BBL (1:100, TKS-1, BioLegend), anti-mouse IL-15Ra (1:50, DNT15Ra, Thermo Fisher Scientific), anti-Flag tag (1:100, L5, BioLegend) for murine IL-7 and IL-12, anti-human β2M (1:50, 2M2, BioLegend), anti-human 4-1BBL (1:100, 5F4, BioLegend), and anti-human IL-12 p40/p70 (1:50, C11.5, BD Biosciences, CA, USA). Cells were washed and analyzed on a NovoCyte^®^ 3000 flow cytometer (Acea Biosciences^™^, Inc., CA, USA). Flow cytometry data were analyzed using FlowJo^™^ software (BD Biosciences). All requests for materials will be promptly reviewed by Rubius Therapeutics to verify whether the request is subject to any intellectual property or confidentiality obligations. Following review, materials can be shared via a material transfer agreement.

### Mouse surrogate mRBC-aAPC preparation

Production of mRBC-H-2K^b^-SIINFEKL (OVA)-4-1BBL, mRBC-OVA-4-1BBL-IL-7, mRBC-OVA-4-1BBL-IL-12, and mRBC-OVA-4-1BBL-IL-15 was based on a method developed using the principles of bioconjugation, commonly referred to as click chemistry^24^. This method uses the specific reaction between an azide group provided by the 6-azidohexanoic acid sulfo-NHS ester (Azido, Click Chemistry Tools, LLC, AZ, USA) and a dibenzocyclooctyne (DBCO) group moiety (Click Chemistry Tools, LLC) or methyltetrazine (MTZ, Click Chemistry Tools, LLC) and a trans-cyclooctene (TCO) group moiety (Click Chemistry Tools, LLC), to generate mRBCs conjugated with recombinant proteins. Recombinant H-2K^b^-SIINFEKL (OVA), murine 4-1BBL (4-1BBL), Fc-murine IL-7 (Fc-mIL-7), Fc-murine IL-12 (Fc-mIL-12), and Fc-human IL-15/IL15Rα fusion (Fc-hIL-15) were produced in Expi293F cells and purified from cell culture supernatant by fast protein liquid chromatography and immobilized metal ion affinity chromatography using an ÄKTA^™^ Pure 25 or ÄKTA^™^ Avant 150 chromatography system (Cytiva, MA, USA) and Ni Sepharose^®^ Excel resin (Cytiva), or protein A affinity purification (MabSelect SuRe^™^ antibody purification resin, Cytiva). The purity and aggregation profile were determined, and the proteins were further purified with HiLoad^®^ 26/600 Superdex^®^ 200 prep grade resin (Cytiva), if needed. Endotoxin levels in protein preparations were determined by Limulus amebocyte lysate assay (Charles River Endosafe^®^ cartridges, Charles River Laboratories) and were low (<10 EU/mg). Following production and purification, OVA, m4-1BBL, Fc-mIL-7, and Fc-mIL-12 were conjugated to a DBCO group moiety, which reacts with the Azido group in the clicking reaction. Fc-hIL-15 was conjugated to a TCO group moiety, which reacts with an MTZ group in the clicking reaction. Excess labeling reagent was removed with Sephadex^™^ G-25 Fine gel filtration resin (Cytiva). All requests for materials will be promptly reviewed by Rubius Therapeutics to verify whether the request is subject to any intellectual property or confidentiality obligations. Following review, materials can be shared via a material transfer agreement.

Blood was collected via a terminal cardiac puncture in mice and filtered through Acrodisc^®^ PSF 25 mm white blood cell syringe filters (Pall Corporation, NY, USA) to remove leukocytes. Cells were then counted and labeled with Azido or Azido together with MTZ. Chemical conjugation of the proteins onto the cells was achieved during overnight incubation. Conjugated cells were extensively washed before formulation and stained with the following antibodies to assess conjugation efficiency: anti-H-2K^b^-OVA (1:100, 25-D1.16, BioLegend), anti-mouse 4-1BBL (1:100, TKS-1, BioLegend), or anti-mouse IgG2a (1:100, RMG2a-62, BioLegend) for the detection of mutated Fc tag on IL-7, IL-12, or IL-15.

To generate mRBC-H-2D^b^-gp100-4-1BBL-IL-12, Azido-labeled mRBCs were incubated with DBCO-IL-12 and DBCO-4-1BBL overnight, followed by incubation with water soluble Biotin DBCO (Click Chemistry Tools, LLC). Biotin copy number was determined using Quantum^™^ Simply Cellular^®^ anti-mouse IgG beads after anti-biotin staining (1:100, BK-1/39, Thermo Fisher Scientific). NeutrAvidin^™^ protein (Thermo Fisher Scientific) was incubated with mRBC-4-1BBL-IL-12-biotin at a 1:1 molar ratio of NeutrAvidin protein and biotin. Biotin-H-2D^b^-gp100 monomer (MBL International Corporation, MA, USA) was then added to bind to the NeutrAvidin protein at a 1:1 molar ratio. Conjugation efficiency was determined after extensive washes and by staining with anti-H-2K^b^/H-2D^b^ antibody (1:100, 28-8-6, BioLegend).

### In vitro CD8^+^ T-cell expansion and activation assay

CD8^+^ T cells were isolated from the spleen and lymph nodes of OT-1 or pmel-1 transgenic mice using the Mouse CD8 + T Cell Isolation Kit (Miltenyi Biotec, Inc., Bergisch Gladbach, Germany). Isolated CD8^+^ T cells were labeled with 1 μM CellTrace^™^ Violet dye (Thermo Fisher Scientific) at 37 °C for 6 min. After quenching with ice-cold fetal bovine serum (FBS, Thermo Fisher Scientific), and extensive washing, labeled OT-1 or pmel-1 cells were resuspended in assay media and plated into each well of a 96-well plate (Corning, PA, USA). Assay media was prepared using Roswell Park Memorial Institute (RPMI) 1640 media with 10% FBS, 100 units/mL penicillin (Thermo Fisher Scientific), 100 μg/mL streptomycin (Thermo Fisher Scientific), 100 μM non-essential amino acids (Thermo Fisher Scientific), 1 mM MEM sodium pyruvate (Thermo Fisher Scientific),10 mM 4-(2-hydroxyethyl)-1-piperazineethanesulfonic acid (HEPES, Thermo Fisher Scientific), and 0.05 mM β-mercaptoethanol (Sigma-Aldrich, Inc.).

RCT-CTRL, mRBC-CTRL, RCT-OVA-4-1BBL, RCT-OVA-4-1BBL-IL-12, and mRBC-OVA-4-1BBL-IL-12 cells were counted, washed, resuspended in assay buffer and plated into wells containing OT-1 cells or pmel-1 cells. Dynabeads^®^ mouse T-Activator CD3/CD28 beads (Thermo Fisher Scientific) were washed, resuspended in assay buffer and incubated with pmel-1 cells as controls. Plates were placed in a humidified incubator at 37 °C. After 4 days, cell culture supernatant was collected and secreted IFNγ was measured using LEGENDplex^™^ MU Th1/Th2 Panel (8-plex) cytokine bead array (BioLegend) according to the manufacturer’s instructions. Cells were processed for flow cytometry after 3–4 days of co-culture.

### Tumor killing assay

For the tumor killing assay, OT-1 cells were cultured 1:10 with RCT-OVA-4-1BBL, RCT-OVA-4-1BBL-IL-7, RCT-OVA-4-1BBL-IL-12, or RCT-OVA-4-1BBL-IL-15 for 3 days. OT-1 cells were then harvested and treated with an Ammonium-Chloride-Potassium (ACK) Lysing Buffer (Thermo Fisher Scientific). Approximately 1 × 10^4^ CellTrace Far Red dye-labeled (Thermo Fisher Scientific) EL4 (control target) or EG7.OVA (target) cells were then incubated with expanded OT-1 cells (effector cells) at a 5:1, 2:1, 1:1, 0.5:1, or 0:1 effector-to-target ratio. After a 22-h incubation period, cells were stained with LIVE/DEAD^™^ Fixable Aqua Dead Cell Stain (Thermo Fisher Scientific) and fixed with 2% paraformaldehyde (Thermo Fisher Scientific) to enumerate live target cells (CellTrace Far Red dye^+^) in each well.

### Adoptive transfer of T cells into mice

The Mouse CD8 + T Cell Isolation Kit was used to isolate CD8^+^ T cells from the spleens and lymph nodes of OT-1 or pmel-1 CD8 transgenic mice. Isolated CD8^+^ T cells were then labeled with 1 μM CellTrace Violet dye at 37 °C for 10 min. For the biodistribution study, isolated OT-1 cells were labeled with 5 μM CellTrace Yellow dye at 37 °C for 8 min. After quenching with ice-cold FBS and extensive washing, 1 × 10^6^ to 2 × 10^6^ labeled T cells were injected intravenously into mice, several hours before the first mRBC-aAPC dose. Antigen-specific donor T cells were tracked in recipient mice using distinct congenic markers (CD45.2 for OT-1 in Pep Boy CD45.1 recipients; CD90.1 for pmel-1 in C57BL/6J CD90.2 recipients).

### EG7.OVA and EL4 subcutaneous tumor model

Pep Boy (CD45.1 B6) mice were injected subcutaneously in the flank with 2 × 10^6^ EG7.OVA at a 1:1 ratio in RPMI 1640 media and Matrigel^®^ matrix (Corning). Tumor growth was measured every 2–3 days. Tumor volume was determined as length (mm) × width (mm^2^) × 0.5. Tumor-bearing mice were randomized when the average tumor volume reached 150–230 mm^3^ and naïve CD8^+^ OT-1 cells were administered to all groups no later than 1 day after randomization by tail vein injection. Several hours after T-cell injection, mice were treated intravenously with PBS (Thermo Fisher Scientific), mRBC-CTRL, or mRBC-aAPC in 200 μL PBS every 3–4 days. For comparison with therapeutic vaccines, a group of mice received 40 nM OVA_241-270_^58^ (AnaSpec^®^, Inc., CA, USA) emulsified in IFA (Sigma-Aldrich). In studies without OT-1 transfer, mRBC-CTRL or mRBC-aAPC treatment started 1 day after subcutaneous injection of 2 × 10^6^ EG7.OVA cells in C57BL/6 mice. For the tumor rechallenge experiment, cured mice were challenged in the alternate flank subcutaneously with 2 × 10^6^ EG7.OVA cells at a 1:1 ratio in RPMI 1640 media and Matrigel matrix. Mice cured following rechallenge with EG7.OVA tumor were then challenged subcutaneously in the shoulder with 1 × 10^5^ EL4 at a 1:1 ratio in RPMI 1640 media and Matrigel matrix. Mice were euthanized after a tumor volume greater than 2000 mm^3^ was reached.

### B16-F10 lung metastasis model

C57BL/6J mice were injected intravenously with 1 × 10^5^ B16-F10 in 200 μL RPMI 1640 media on day 0. The next day, all groups were intravenously administered 2 × 10^6^ pmel-1 cells, followed by intravenous administration of mRBC-CTRL or mRBC-gp100-4-1BBL-IL-12 on days 1, 4, and 8. One group of mice received 100 μg anti-PD-1 (*InVivo*Mab™ anti-mouse PD-1 clone RMP1-14, Bio X Cell, Inc., NH, USA) intraperitoneally on days 1, 4, and 8 in addition to mRBC-CTRL treatment. On day 14, the animals were euthanized. The lungs were perfused with ice-cold PBS through the right atrium and removed, and the lobes were teased apart with small forceps in a Petri dish. The left lobe was processed immediately for immune cell phenotyping. The remaining four lobes were fixed in 10% buffered formalin (VWR International, LLC, PA, USA) for 24 h. Metastases were visualized and quantified under the microscope by an operator blinded to the treatments. All mice requiring euthanasia or found dead for reasons other than tumor burden were excluded from analysis.

### Immune profiling in mice and flow cytometry

Single-cell suspensions were prepared from blood, spleens, lymph nodes, lungs, and tumors. Blood was collected by submandibular bleed or cardiac puncture in BD Microtainer^®^ blood collection tubes with K_2_EDTA (BD Biosciences) and the tubes were kept on ice until analysis. For flow cytometry, 50 μL of blood was mixed with 550 μL of Alfa Aesar^™^ RBC Lysis Buffer for mouse RBCs (Thermo Fisher Scientific) in a 96-well deep well plate (VWR International, LLC) and incubated at room temperature for 15 min to lyse RBCs. All cells were transferred to a round bottom 96-well plate (Corning) after pelleting for staining. Tumors or lungs were digested in gentleMACS^™^ C-tubes (Miltenyi Biotec, Inc.) using the murine Tumor Dissociation Kit or the murine Lung Dissociation Kit (Miltenyi Biotec, Inc.), respectively. Cell suspensions were generated using a gentleMACS Octo Dissociator with Heaters instrument (Miltenyi Biotec, Inc.) according to the manufacturer’s instructions. Digested tissue was then filtered through a 70 µm cell strainer and washed and resuspended with PBS containing 0.1% bovine serum albumin (PBSA; Cytiva) for staining.

For flow cytometry, cells were resuspended in a staining mixture containing Fc block (1:100 from 1 mg/mL working stock, anti-mouse CD16/32, clone 2.4G2, Bio X Cell, Inc.), directly conjugated antibodies, and LIVE/DEAD Fixable Aqua Dead Cell Stain or Zombie NIR^™^ dye (BioLegend) in PBSA and stained for 30 min on ice. For detecting endogenous OVA-specific T cells by tetramer staining, cells were incubated with H-2K^b^-SIINFEKL tetramer (1:20, MBL International Corporation) with Fc block at room temperature for 10 min before surface staining. Samples were washed twice with PBSA and resuspended in PBSA for flow cytometry. To measure intracellular expression, eBioscience™ Cell Stimulation Cocktail (Thermo Fisher Scientific), containing phorbol 12-myristate 13-acetate (PMA), ionomycin, brefeldin A, and monensin, was incubated with single-cell suspensions from tumors for 3–4 h at 37 °C. After incubation, cells were washed, surface stained, fixed, and permeabilized using the eBioscience Foxp3/Transcription Factor Staining Buffer Set (Thermo Fisher Scientific), before being stained for intracellular or intranuclear marker expression according to the manufacturer’s instructions.

The following fluorochrome-conjugated antibodies were purchased from BioLegend or BD Biosciences: anti-CD44 (1:200, 1M7), anti-CD62L (1:200, MEL-14), anti-CD45.2 (1:200, 104), anti-CD8 (1:200, 53-6.7), anti-CD122 (1:100, TM-β1), anti-IFNγ (1:100, XMG1.2), anti-granzyme B (1:100, GB11), anti-CD45.1 (1:200, A20), anti-CD90.1 (1:200, OX-7), anti-CD90.2 (1:200, 53-2.1), anti-Ki67 (1:200, B56), anti-TNFα (1:100, MP6-XT22), anti-IL-2 (1:100, JES6-5H4), anti-NK1.1 (1:200, PK136), anti-CD3 (1:200, 145-2C11), anti-CD4 (1:200, GK1.5), anti-Foxp3 (1:100, FJK-16s), anti-I-A^b^ (1:400, AF6-120.1), anti-CD25 (1:100, PC61), anti-F4/80 (1:100, T45-2342), anti-CD11b (1:200, M1/70), anti-Ly6C (1:200, HK1.4), and anti-Ly6G (1:200, 1A8). Flow cytometry data were acquired on a BD LSRFortessa^™^ flow cytometer (BD Biosciences) with BD FACSDiva™ v8.0.2 software, or a Novocyte 3000 flow cytometer with NovoExpress 1.4.1, and analyzed using FlowJo 10.5.3 software.

### TCR variable beta chain sequencing

Genomic DNA (gDNA) was extracted from 50–60 μL of anticoagulated blood 5–10 min after OT-1 transfer (pre-treatment or pre-rechallenge) or post treatment/tumor rechallenge using DNeasy^®^ Blood and Tissue Kit (Qiagen^®^ Inc., MD, USA) according to the manufacturer’s instructions. Single-cell suspensions were extracted from whole tumors, similar to the tissue processing for flow cytometry described above, and gDNA was extracted from one quarter of the tumor single-cell suspensions using the DNeasy Blood and Tissue Kit (Qiagen Inc.) according to the manufacturer’s instructions. Immunosequencing of the CDR3 regions of mouse TCRβ chains was performed using the immunoSEQ^®^ Assay (Adaptive Biotechnologies Corporation, Seattle, WA). In brief, extracted DNA was amplified in a bias-controlled multiplex polymerase chain reaction (PCR), followed by high-throughput sequencing. Sequences were then collapsed and filtered in order to identify and quantitate the absolute abundance of each unique TCRβ CDR3 region for further analysis with an Adaptive ImmunoSEQ analyzer v3.0^59–61^.

### Safety measurements

Pep Boy (CD45.1 B6) mice that were treated with 1 × 10^6^ naïve OT-1 cells, or left untreated, on day 0, were dosed with 1 × 10^9^ mRBC-CTRL or a dose titration of mRBC-OVA-4-1BBL-IL-12 (1 × 10^9^, 3 × 10^8^) on days 0, 4, 7, and 11. One mouse found dead of uncertain relationship to treatment was excluded from the analysis. Body weight changes compared with day 0 were determined every day, except for weekends. Spleen weight and liver weight were determined on days 12 and 25. Plasma IFNγ, IL-10, TNFα, IL-6, and IL-1β levels were determined using the LEGENDplex Mouse Inflammation Panel (13-plex) multiplex assay (BioLegend). Serum ALT, aspartate transaminase (AST), and alkaline phosphatase (ALP) levels on days 12 and 25 were determined using a Beckman Coulter^™^ AU680 Chemistry Analyzer (Beckman Coulter, Inc., CA, USA). Hematocrit, hemoglobin, and platelet levels on day 12 and 25 were determined with whole blood samples by a Sysmex XT-2000iV™ automated hematology analyzer (Sysmex, Norderstedt, Germany). To determine liver inflammation and macrophage infiltration, formalin-fixed liver samples collected on days 12 and 25 were paraffin embedded, sectioned, and stained with hematoxylin and eosin (H&E) or with anti-mouse F4/80 antibody (1:100, clone BM8, Thermo Fisher Scientific). H&E-stained slides were scored blinded by a board-certified pathologist. Ten high-power fields (HPFs) were randomly selected, and liver perivascular inflammation was scored as follows: 0 = inflammation is not present, 1 = minimal inflammation, 2 = mild inflammation, 3 = moderate inflammation, and 4 = severe inflammation. A single score was assigned based on the 10 HPFs. These scores were analyzed by the non-parametric Kruskai–Wallis test. Liver macrophage infiltration was quantified using HALO^™^ image analysis software V2.2.1870.44 with High Plex FL module V2.0 (Indica Labs, NM, USA), with specific thresholds set to identify the F4/80-positive cells present in the tissue section.

### Biodistribution analyses

mRBC-CTRL or mRBC-OVA-4-1BBL-IL-12 cells were incubated with 10 μM CellTrace Far Red dye at room temperature for 6 min followed by quenching with FBS and extensive washing before dosing. Pep Boy (CD45.1 B6) mice were dosed with 1 × 10^9^ CellTrace Far-Red dye-labeled mRBC-CTRL or mRBC-OVA-4-1BBL-IL-12. At 1 h or 17 h post mRBC-aAPC dosing, mice were euthanized and perfused with PBS. One quarter of the spleen, the whole mandibular lymph node (LN), one of the mesenteric LNs, the bottom half of the left lung lobe, a 5-mm^3^ portion of liver, the bottom half of the heart, a 5-mm^3^ portion of brain, a 5-mm^3^ portion of large intestine, a whole kidney, an ovary, and the testes were frozen in Tissue-Tek^®^ Cryomold^®^ plastic molds (VWR International, LLC) containing Tissue-Tek O.C.T. Compound (VWR International, LLC) in a liquid nitrogen bath. Frozen tissues in O.C.T. Compound were stored at −80 °C before preparation of 7 μM sections that were placed onto slides. Slides were fixed with 4% formalin (Sigma-Aldrich), washed, and incubated with Hoechst dye (Thermo Fisher Scientific). After washing, coverslips were mounted on slides with ProLong^™^ Gold Antifade Mountant (Thermo Fisher Scientific) and scanned on a 3DHISTECH PANNORAMIC™ scanner v2.1.1.10094 RTM (Thermo Fisher Scientific). Images were analyzed for mRBC density per tissue area using HALO image analysis software V2.2.1870.44 with High Plex FL module V2.0. Samples that were not sectioned or stained properly were excluded from analysis and noted in the source data.

### Spleen interaction analyses

CD45.1 Pep Boy mice were transferred with 2 × 10^6^ naïve CellTrace Yellow dye-labeled OT-1 cells before dosing with 1 × 10^9^ CellTrace Far Red dye-labeled mRBC-CTRL, or mRBC-OVA-4-1BBL-IL-12 cells. At 1 h or 17 h post mRBC dosing, mice were euthanized and perfused with PBS. Half of each spleen was processed into a single-cell suspension for flow cytometry analyses without RBC lysis to determine the amount of mRBC that formed doublets with OT-1 cells. One quarter of each spleen was frozen in Tissue-Tek O.C.T. Compound. Frozen tissues in Tissue-Tek O.C.T. Compound were stored at −80 °C before preparation of 7 μM sections that were placed onto slides. Slides were fixed with 4% formalin, washed, blocked with Background Sniper blocking reagent (Biocare Medical, LLC, CA, USA), and stained with hamster anti-mouse-CD31 antibody (1:250, clone 2H8, MilliporeSigma, MA, USA). Slides were washed, stained with anti-hamster antibody conjugated with AF488 (1:125, Jackson ImmunoResearch Laboratories, Inc., PA, USA), and incubated with Hoechst dye (Thermo Fisher Scientific). After washing, coverslips were mounted on slides with ProLong™ Gold Antifade Mountant (Thermo Fisher Scientific) and scanned on an Aperio^™^ ScanScope^®^ FL scanner with ScanScope Console v102.0.0.33 (Leica Microsystems, Inc., IL, USA). The percentage of OT-1 cells that colocalized with mRBCs was determined using the HALO image analysis software with Object Colocalization FL module V1.0. Confocal images of spleen sections were obtained using a Leica TCS SP8 STED 3X confocal microscope (Leica Microsystems, Inc.) and LAS X Life Science 3.5.5.19976 software (Leica Microsystems, Inc.).

### In vitro live cell imaging

CD8^+^ T cells were isolated from the spleen and lymph nodes of OT-1 mice and incubated with Dynabeads mouse T-Activator CD3/CD28 beads at a 1:1 ratio for 2 days to upregulate 4-1BB expression. The beads were removed before incubating activated OT-1 cells with AlexaFluor 488^®^ dye (AF488)-conjugated Fab of anti-mouse TCRβ antibody clone H57-597 (250 μg/mL, Bio X Cell). mRBC-OVA-4-1BBL-IL-12 was conjugated with DyLight^™^ 650 dye (DL650)-labeled 4-1BBL protein, which has been determined to be similarly functional to unlabeled 4-1BBL protein. mRBC-CTRL cells were labeled with 10 μM CellTrace Far Red dye at room temperature for 6 min. Labeled mRBC-CTRL or mRBC-OVA-4-1BBL-IL-12 cells were immobilized on Nunc^™^ Lab-Tek^™^ II 8-chambered coverglass (Thermo Fisher Scientific) that were pre-coated with anti-TER-119 antibody (25 μg/mL, Thermo Fisher Scientific). Around 10 min after adding AF488-H57-labeled activated OT-1 cells to the immobilized mRBC-CTRL or mRBC-OVA-4-1BBL-IL-12 cells at 37 °C, images were acquired on a Leica TCS SP8 STED 3X confocal microscope with a ×100 objective every 10 s for 15 min using LAS X Life Science 3.5.5.19976 software. Mander’s colocalization coefficient was determined using the EzColocalization plugin^62^ in the ImageJ 2.0.0-rc-69/1.52p software (Java 1.8.0_172, 64 bit) to quantify percent above the background OT-1 signal, which overlaps with the percent above the background mRBC signal in each frame of the live cell imaging video.

### TCR transduction of primary human T cells and co-culture assay with engineered RBCs

CD8^+^ T cells from human HLA-A*02:01-positive donors (Cellero^™^, MA, USA), collected under Advarra IRB approval and informed consent, were stimulated with Dynabeads human T-Activator CD3/CD28 beads (Thermo Fisher Scientific) at a 1:1 ratio in X-VIVO^™^15 medium containing 100 IU/mL human IL-2 (PeproTech, NJ, USA). The next day, the beads were removed and activated CD8^+^ T cells were transduced with lentivirus encoding HPV E7_11-19_-specific TCR in X-VIVO 15 media containing 100 IU/mL human IL-2. Culture media was changed 1, 4, and 8 days after transduction. On day 10, 8 × 10^4^ untransduced T cells or TCR-engineered primary CD8^+^ T cells with ~20% TCR expression were incubated with 3.2 × 10^5^ RTX-321 or control RBCs in 200 μL X-VIVO 15 media without human IL-2. At 2 h, cells were harvested, surface stained, fixed, and permeabilized using Foxp3/Transcription Factor Staining Buffer Set (Thermo Fisher Scientific), and stained for Nur77 expression. On day 1, cells were treated with brefeldin A (BioLegend) for 4 h before harvest and were stained with a similar protocol for CD69 and granzyme B expression. On day 5, cells were harvested for flow cytometry analyses of HPV E7_11-19_ tetramer^+^ (1:10, MBL International Corporation) CD8^+^ T-cell numbers and live CD8^+^ T-cell phenotype. The following antibodies were utilized after dead cell exclusion by LIVE/DEAD Fixable Aqua Dead Cell Stain: anti-CD8 (1:200, RPA-T8, BioLegend), anti-mouse TCRβ (1:100, H57-597, BioLegend), anti-Nur77 (1:100, 12.14, Thermo Fisher Scientific), anti-CD69 (1:200, FN50, BioLegend), anti-4-1BB (1:200, 4B4-1, BioLegend), anti-CCR7 (1:200, G043H7, BioLegend), anti-CD45RO (1:200, UCHL1, BioLegend), and anti-granzyme B (1:200, QA16A02, BioLegend). Flow cytometry data were acquired on a BD LSRFortessa flow cytometer with BD FACSDiva v8.0.2 and analyzed using FlowJo 10.5.3 software. After 5 days, cell culture supernatant was collected, and secreted IFNγ was measured using the LEGENDplex Human CD8/NK Panel (13-plex) multiplex assay (BioLegend). The assay was performed according to the manufacturer’s instructions.

### Statistics

Graphs were made and statistical analyses were performed using Prism software v8.4.2 (GraphPad Software, Inc., CA, USA). Data were expressed as mean ± standard deviation. Analysis of two groups was performed by a two-tailed, unpaired Student’s *t* test where applicable. For analyses of three or more groups, a one-way analysis of variance (ANOVA) test was performed with Dunnett’s multiple comparison test at each time point compared to controls. Biodistribution significance was determined by one-way ANOVA with Tukey’s multiple comparison test within each tissue type. T-cell expansion post tumor rechallenge was determined by one-way ANOVA with Dunnett’s multiple comparison test compared to before challenge. Body weight and plasma cytokine levels over time were analyzed by repeated measure two-way ANOVA with Tukey’s multiple comparisons test at each time point, and statistical significance was reported for each time point compared to similarly treated mRBC-CTRL. Tetramer+ CD8 + T cells in the blood over time were analyzed by repeated measure two-way ANOVA with Dunnett’s multiple comparison test compared to mRBC-CTRL after log transformation of data points (data do not follow normal distribution as determined by Shapiro–Wilk test). Statistical differences in survival were determined by log rank (Mantel-Cox) analyses. *P* < 0.05 was considered statistically significant.

### Reporting summary

Further information on research design is available in the Nature Research Reporting Summary linked to this article.

## Supplementary information

Supplementary Information

Descriptions of Additional Supplementary Files

Supplementary movie 1

Supplementary movie 2

Reporting Summary

## Data Availability

The TCR sequencing data generated in this study have been deposited in the Gene Expression Omnibus (GEO) database under accession code GSE168826. Source data are available as a source data file. The authors declare that all data supporting the findings of this study are available within the paper and its supplementary information files or available from the authors upon request. All requests for materials will be promptly reviewed by Rubius Therapeutics to verify whether the request is subject to any intellectual property or confidentiality obligations. Following review, materials can be shared via a material transfer agreement by contacting the corresponding author. Source data are provided with this paper.

## Source data

Source Data
