# Supplementary material for: Engineered red blood cells as an off-the-shelf allogeneic anti-tumor therapeutic
Source: Nat Commun. 2021 May 11;12:2637. doi: 10.1038/s41467-021-22898-3 (PMC8113241; doi:10.1038/s41467-021-22898-3)
Supplement: Supplementary file 1 — Supplementary Information [file 41467_2021_22898_MOESM1_ESM.pdf]

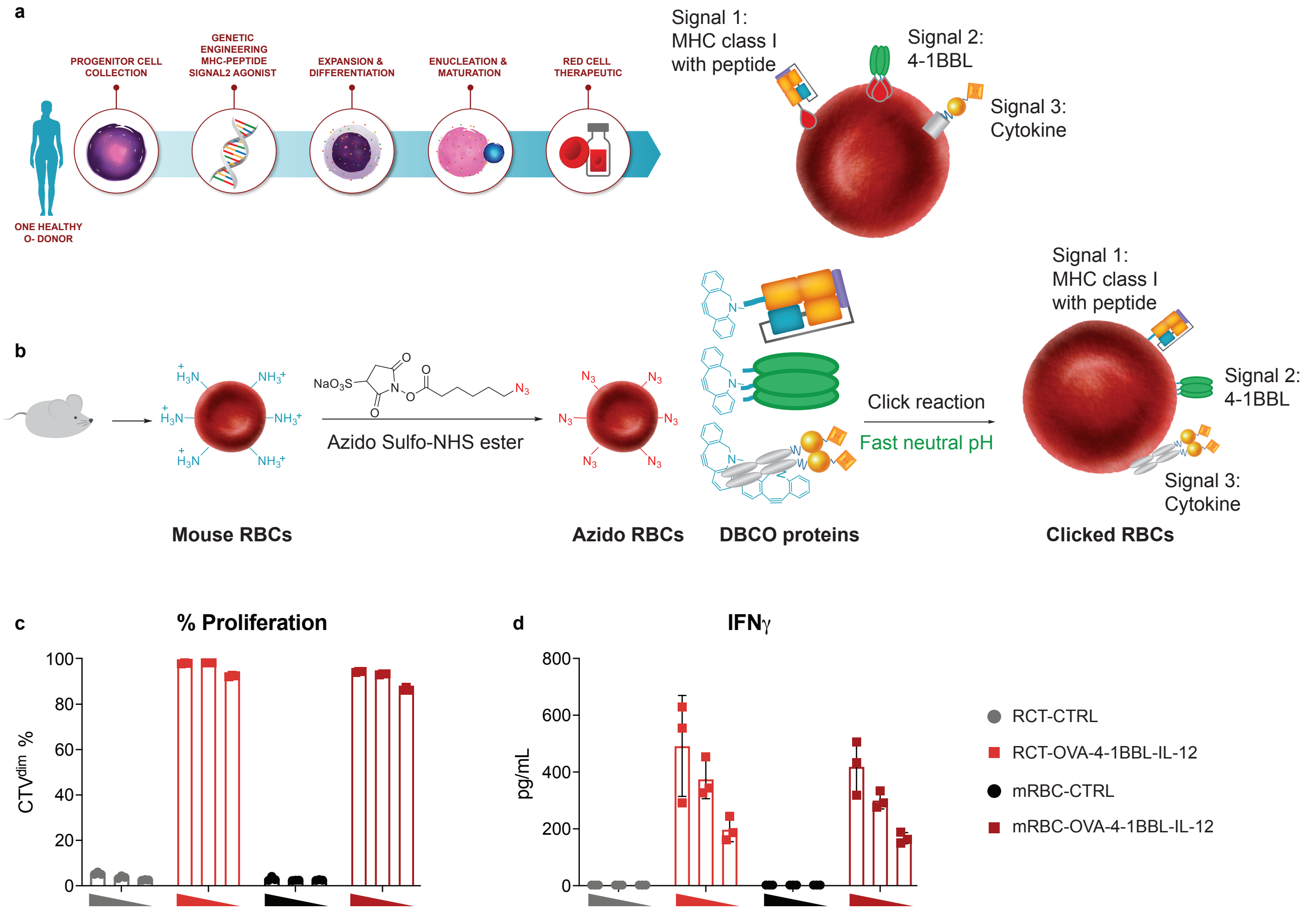
a
PROGENITOR CELL COLLECTION
GENETIC ENGINEERING MHC-PEPTIDE SIGNAL2 AGONIST
EXPANSION & DIFFERENTIATION
ENUCLEATION & MATURATION
RED CELL THERAPEUTIC
ONE HEALTHY O- DONOR
Signal 1: MHC class I with peptide
Signal 2: 4-1BBL
Signal 3: Cytokine
b
Azido Sulfo-NHS ester
Click reaction
Fast neutral pH
Signal 1: MHC class I with peptide
Signal 2: 4-1BBL
Signal 3: Cytokine
Mouse RBCs
Azido RBCs
DBCO proteins
Clicked RBCs
c
% Proliferation
CTV^dim %
d
IFNγ
pg/mL
RCT-CTRL
RCT-OVA-4-1BBL-IL-12
mRBC-CTRL
mRBC-OVA-4-1BBL-IL-12

Supplementary Fig. 1 RCT-aAPCs and murine surrogate mRBC-aAPCs. a, Generation of RCT-aAPCs that express peptide-MHC class I complex fused to an antigenic peptide as signal 1, 4-1BBL as signal 2, and a cytokine as signal 3. **b**, Generation of mRBC-aAPCs using a click chemistry reaction to conjugate on the surface of mRBC peptide-MHC class I complex as signal 1, 4-1BBL as signal 2, and a cytokine as signal 3. **c-d**, CellTrace Violet dye-labeled OT-1 cells ($1x10^5$ per well, n=3) were co-cultured with RCT-OVA-4-1BBL-IL-12, RCT-CTRL, mRBC-CTRL, or mRBC-OVA-4-1BBL-IL-12 at a 1:9, 1:3 or 1:1 ratio. % proliferation in OT-1 cells (**c**), and IFNγ concentration in supernatant (**d**) on day 4. Data are depicted as mean ± s.d and are representative of two independent experiments. Source data are provided as a Source Data file.

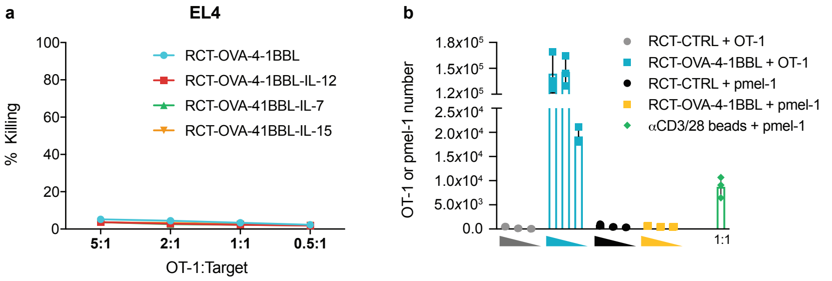
a
EL4
% Killing
100
80
60
40
20
0
RCT-OVA-4-1BBL
RCT-OVA-4-1BBL-IL-12
RCT-OVA-41BBL-IL-7
RCT-OVA-41BBL-IL-15
5:1
2:1
1:1
0.5:1
OT-1:Target
b
OT-1 or pmel-1 number
1.8x10^5
1.5x10^5
1.2x10^5
2.5x10^4
2.0x10^4
1.5x10^4
1.0x10^4
5.0x10^3
0.0
RCT-CTRL + OT-1
RCT-OVA-4-1BBL + OT-1
RCT-CTRL + pmel-1
RCT-OVA-4-1BBL + pmel-1
αCD3/28 beads + pmel-1
1:1

Supplementary Fig. 2 Antigen-specificity of RCT-aAPCs. a, EL4 percent killing by RCT-activated OT-1 $CD8^+$ T cells (n=3). **b**, OT-1 or pmel-1 numbers after 3 days co-incubation with a dose titration ($1x10^6$, $3x10^5$, or $1x10^5$) of either RCT-CTRL or RCT-OVA-4-1BBL (n=3) with $1x10^5$ T cells, or $1x10^5$ anti-CD3/CD28 beads with $1x10^5$ T cells. Data are depicted as mean ± s.d and are representative of two (**a**) or one (**b**) independent experiments. Source data are provided as a Source Data file.

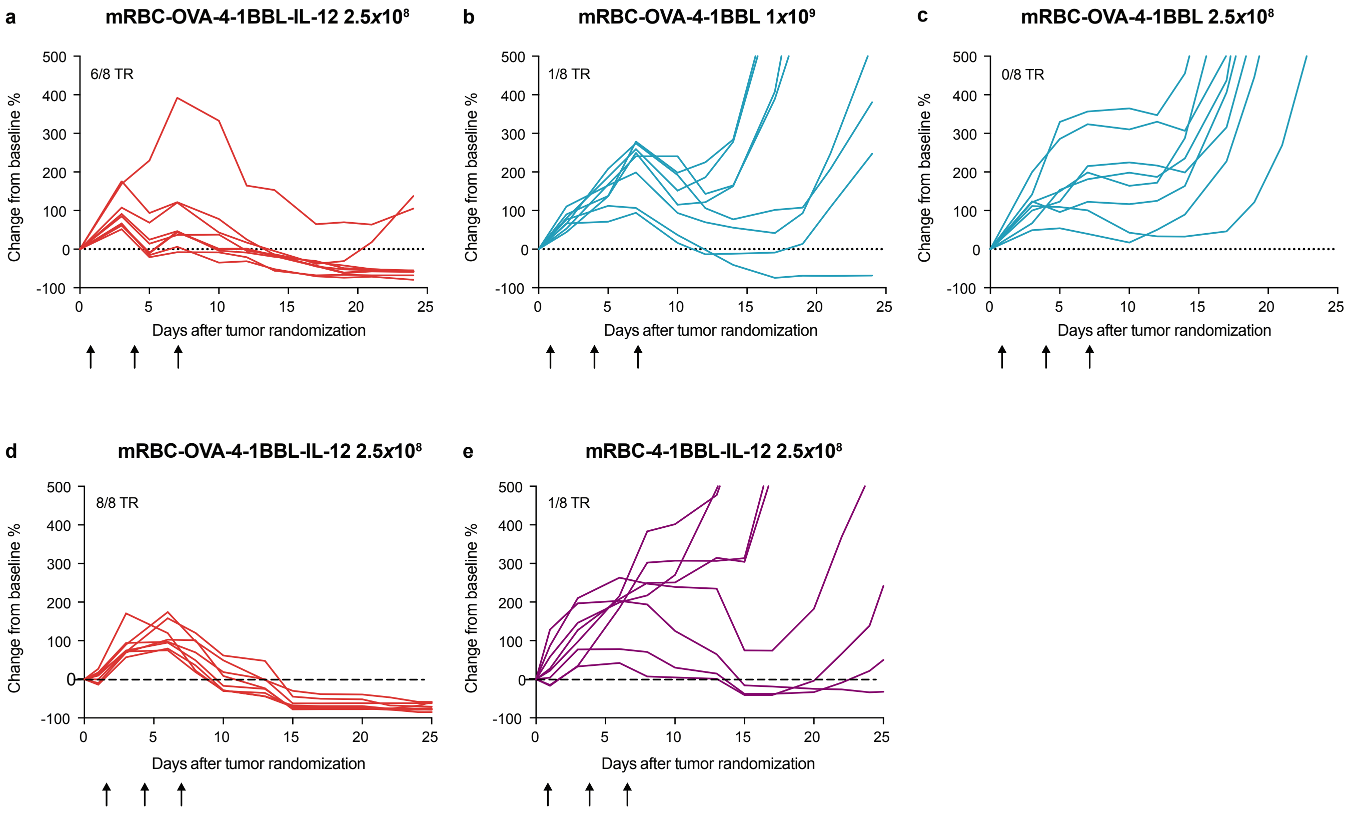

a
mRBC-OVA-4-1BBL-IL-12 2.5x10^8
6/8 TR
b
mRBC-OVA-4-1BBL 1x10^9
1/8 TR
c
mRBC-OVA-4-1BBL 2.5x10^8
0/8 TR
d
mRBC-OVA-4-1BBL-IL-12 2.5x10^8
8/8 TR
e
mRBC-4-1BBL-IL-12 2.5x10^8
1/8 TR
Change from baseline %
Days after tumor randomization

Supplementary Fig. 3 mRBC-OVA-4-1BBL-IL-12 promotes tumor regressions in EG7.OVA tumor-bearing mice. CD45.1 Pep Boy mice were injected subcutaneously with 2*x*10^6 EG7.OVA cells. When the tumors reached a volume of ~150 mm^3, the animals were randomized (n=8) and dosed on day 1 post randomization with 1*x*10^6 naïve OT-1 cells. The animals were then dosed on days 1, 4 and 7 with mRBC. **a**–**c**, Tumor regression (TR, reduction of tumor volume on day 24 compared to tumor volume at randomization) in 2.5*x*10^8 mRBC-OVA-4-1BBL-IL-12 (**a**), 1*x*10^9 mRBC-OVA-4-1BBL (**b**), or 2.5*x*10^8 mRBC-OVA-4-1BBL (**c**) treated mice. In a separate study, CD45.1 Pep Boy mice were injected subcutaneously with 2*x*10^6 EG7.OVA cells. When the tumors reached a volume of ~230 mm^3, the animals were randomized (n=8) and dosed on day 1 post-randomization with 1*x*10^6 naïve OT-1 cells. The animals were then dosed on days 1, 4 and 7 with mRBC. **d**-**e,** TR of 2.5*x*10^8 mRBC-OVA-4-1BBL-IL-12 (**d**) or 2.5*x*10^8 mRBC-4-1BBL-IL-12 (**e**) treated mice. Results are representative of five (**a**,**d**), three (**b**), or two (**c**, **e**) independent experiments. Source data are provided as a Source Data file.

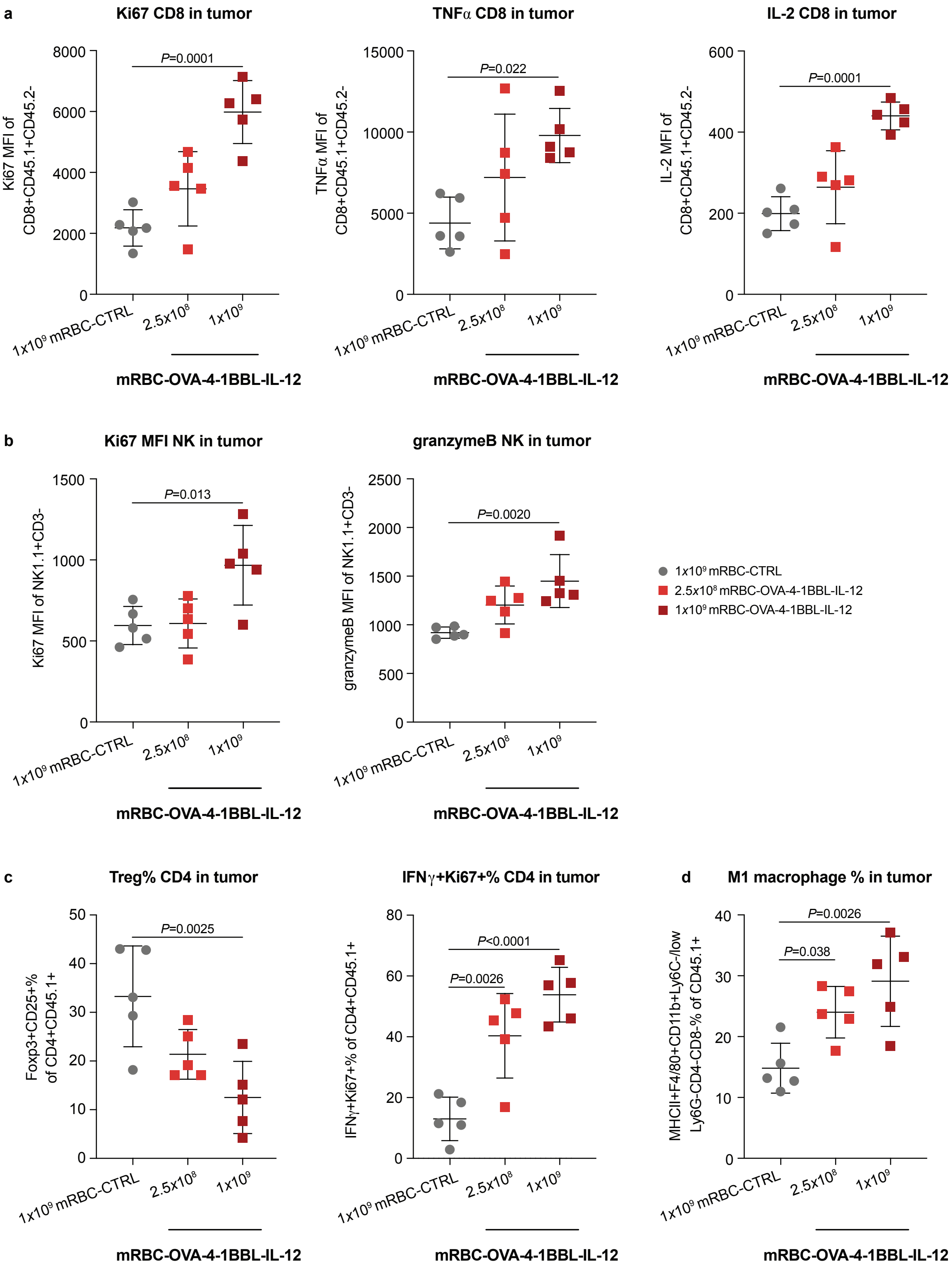

a
Ki67 CD8 in tumor
Ki67 MFI of CD8+CD45.1+CD45.2-
P=0.0001
TNFα CD8 in tumor
TNFα MFI of CD8+CD45.1+CD45.2-
P=0.022
IL-2 CD8 in tumor
IL-2 MFI of CD8+CD45.1+CD45.2-
P=0.0001
b
Ki67 MFI NK in tumor
Ki67 MFI of NK1.1+CD3-
P=0.013
granzymeB NK in tumor
granzymeB MFI of NK1.1+CD3-
P=0.0020
1x10⁹ mRBC-CTRL
2.5x10⁸ mRBC-OVA-4-1BBL-IL-12
1x10⁹ mRBC-OVA-4-1BBL-IL-12
c
Treg% CD4 in tumor
Foxp3+CD25+% of CD4+CD45.1+
P=0.0025
IFNγ+Ki67+% CD4 in tumor
IFNγ+Ki67+% of CD4+CD45.1+
P<0.0001
P=0.0026
d
M1 macrophage % in tumor
MHCII+F4/80+CD11b+Ly6C-/low Ly6G-CD4-CD8-% of CD45.1+
P=0.0026
P=0.038
1x10⁹ mRBC-CTRL
2.5x10⁸
1x10⁹
mRBC-OVA-4-1BBL-IL-12

Supplementary Fig. 4 mRBC-OVA-4-1BBL-IL-12 promoted general anti-tumor immune effects. CD45.1 Pep Boy mice were inoculated subcutaneously with $2x10^6$ EG7.OVA cells. When the tumors reached a volume of ~175 mm^3, the animals were randomized (n=5) and treated with $1x10^6$ naïve OT-1 cells. After, $1x10^9$ mRBC-CTRL or a dose titration of mRBC-OVA-4-1BBL-IL-12 ($1x10^9$, $2.5x10^8$) was administered on days 0 and 3. Mice were sacrificed on day 7. **a**, Ki67, TNFα, and IL-2 per cell expression in tumor infiltrating endogenous $CD8^+$ T cells; **b**, Ki67, and granzyme B per cell expression in tumor-infiltrating NK cells; **c**, Treg% and $IFN\gamma^+Ki67^+$% in tumor infiltrating $CD4^+$ T cells; and **d**, M1 macrophage % in tumor infiltrating leukocytes. Data are depicted as mean ± s.d. and are representative of two independent experiments. One-way ANOVA compared to mRBC-CTRL. Source data are provided as a Source Data file.

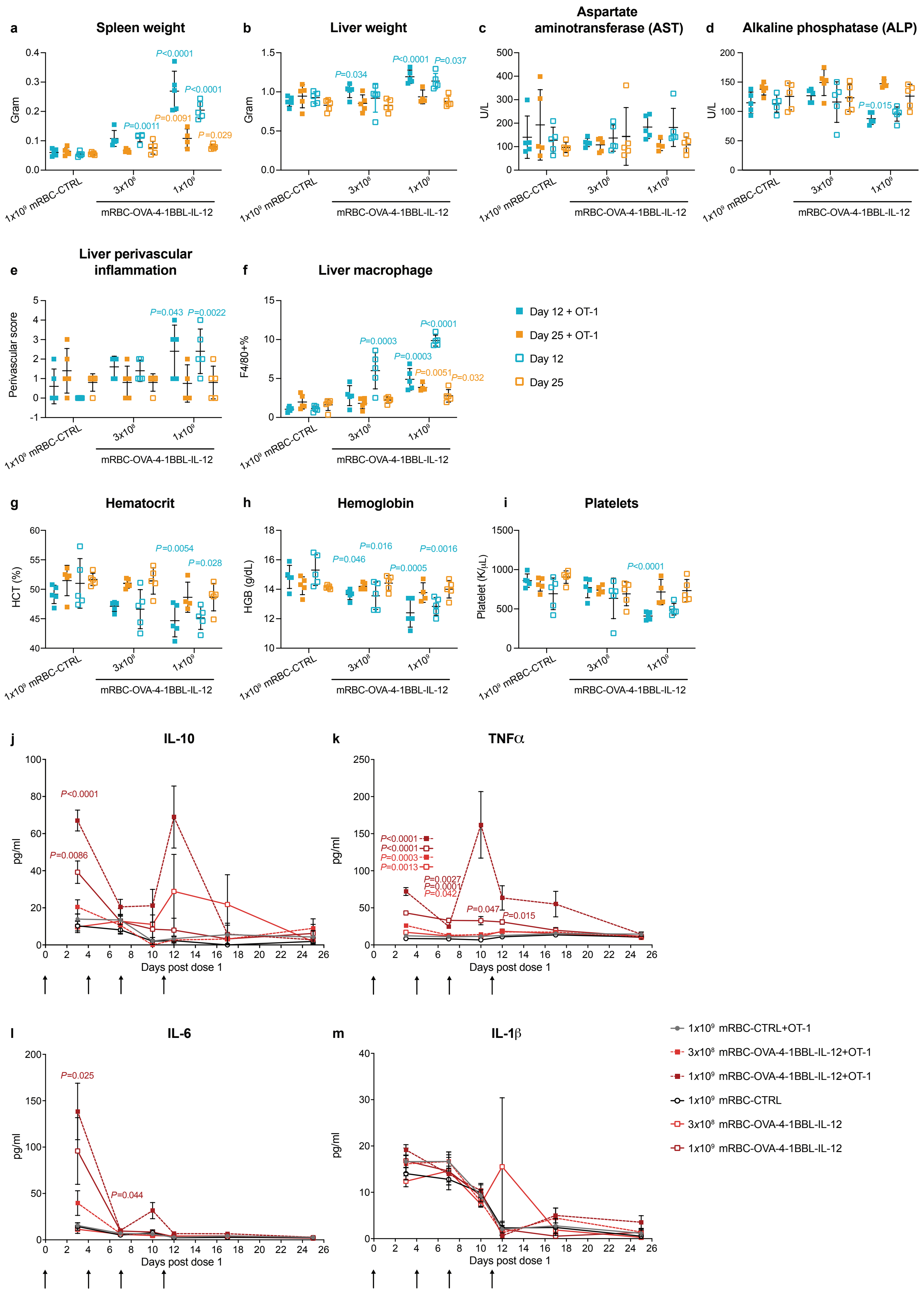

a
Spleen weight
Gram
P<0.0001
P<0.0001
P=0.0011
P=0.0091
P=0.029
b
Liver weight
Gram
P=0.034
P<0.0001
P=0.037
c
Aspartate aminotransferase (AST)
U/L
d
Alkaline phosphatase (ALP)
U/L
P=0.015
1x10^9 mRBC-CTRL
3x10^8
1x10^9
mRBC-OVA-4-1BBL-IL-12
e
Liver perivascular inflammation
Perivascular score
P=0.043
P=0.0022
f
Liver macrophage
F4/80+%
P<0.0001
P=0.0003
P=0.0003
P=0.0051
P=0.032
Day 12 + OT-1
Day 25 + OT-1
Day 12
Day 25
g
Hematocrit
HCT (%)
P=0.0054
P=0.028
h
Hemoglobin
HGB (g/dL)
P=0.016
P=0.0016
P=0.046
P=0.0005
i
Platelets
Platelet (K/μL)
P<0.0001
j
IL-10
pg/ml
P<0.0001
P=0.0086
Days post dose 1
k
TNFα
pg/ml
P<0.0001
P<0.0001
P=0.0003
P=0.0013
P=0.0027
P=0.0001
P=0.042
P=0.047
P=0.015
Days post dose 1
l
IL-6
pg/ml
P=0.025
P=0.044
Days post dose 1
m
IL-1β
pg/ml
Days post dose 1
1x10^9 mRBC-CTRL+OT-1
3x10^8 mRBC-OVA-4-1BBL-IL-12+OT-1
1x10^9 mRBC-OVA-4-1BBL-IL-12+OT-1
1x10^9 mRBC-CTRL
3x10^8 mRBC-OVA-4-1BBL-IL-12
1x10^9 mRBC-OVA-4-1BBL-IL-12

Supplementary Fig. 5 mRBC-OVA-4-1BBL-IL-12 was well tolerated. CD45.1 Pep Boy mice (n=10) were treated with $1x10^6$ naïve OT-1 cells or left untreated, and dosed with either $1x10^9$ mRBC-CTRL or a dose titration of mRBC-OVA-4-1BBL-IL-12 ($1x10^9$ or $3x10^8$) on days 0, 4, 7 and 11. n=5 were sacrificed on Day 12 with n=5 measured for time points post day 12 for all groups except for n=4 for mRBC-OVA-4-1BBL-IL-12+OT-1. **a**, Spleen weight; **b**, liver weight; **c**, serum aspartate aminotransferase (AST) levels; **d**, serum alkaline phosphatase (ALP) levels; **e**, liver perivascular inflammation score; **f**, liver macrophage infiltration; **g**, hematocrit levels; **h**, hemoglobin levels; **i**, platelet levels on days 12 and 25 (**a**-**i**: n=5 for all groups except for n=4 for mRBC-OVA-4-1BBL-IL-12+OT-1 on day 25). **j**, Plasma IL-10 levels; **k**, plasma TNFα levels; **l**, plasma IL-6 levels; **m**, plasma IL-1β levels over time (**j**-**m**: n=10 for days 3 and 7, n=5 for days 10, 12, 17 and 25 except for n=4 for mRBC-OVA-4-1BBL-IL-12+OT-1 on days 17 and 25). One-way ANOVA (**a**, **b**, **c**, **d**, **f**, **g**, **h**, **i**), one-way ANOVA nonparametric Kruskal-Wallis test with Dunn's multiple comparisons test (**e**), two-way ANOVA (**j**, **k**, **l**, **m**) compared to mRBC-CTRL. Data are depicted as mean ± s.d. and are representative of two independent experiments. Source data are provided as a Source Data file.

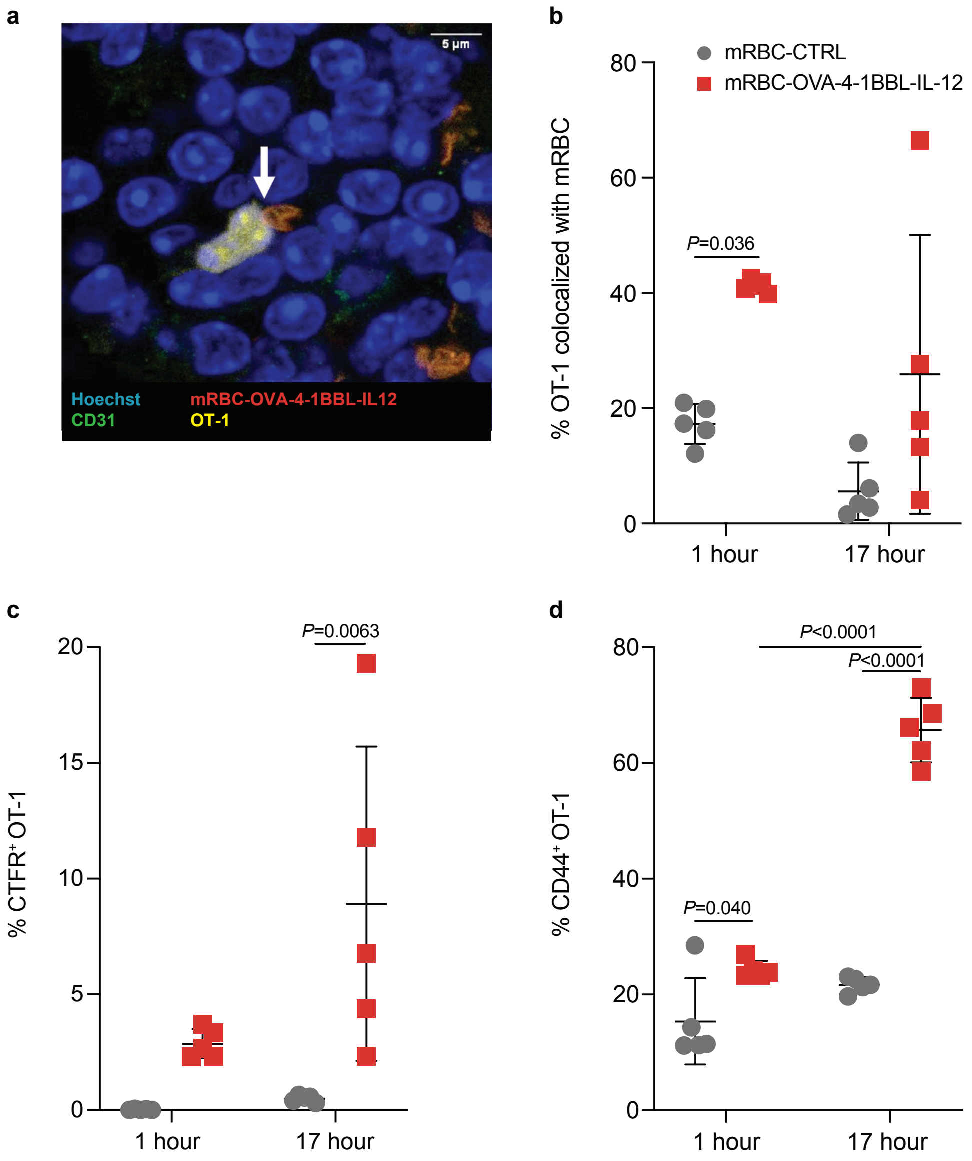
a
5 µm
Hoechst
CD31
mRBC-OVA-4-1BBL-IL12
OT-1
b
mRBC-CTRL
mRBC-OVA-4-1BBL-IL-12
% OT-1 colocalized with mRBC
P=0.036
1 hour
17 hour
c
% CTFR⁺ OT-1
P=0.0063
1 hour
17 hour
d
% CD44⁺ OT-1
P<0.0001
P<0.0001
P=0.040
1 hour
17 hour

Supplementary Fig. 6 mRBC-OVA-4-1BBL-IL-12 interacts with OT-1 cells in the spleen. CD45.1 Pep Boy mice (n=5) were transferred with 2*x*10^6 naïve CellTrace Yellow dye-labeled OT-1 cells before dosing with 1*x*10^9 CellTrace Far Red dye-labeled mRBC-CTRL, or mRBC-OVA-41BBL-IL12. One hour or 17 hours post mRBC injection, mice were sacrificed. **a**-**b**, immunofluorescence analyses of spleen. Representative confocal image of OT-1 and mRBC-OVA-4-1BBL-IL12 interaction 1-hour post dose (**a**) and quantification of % OT-1 co-localized with mRBCs (**b**). One-way ANOVA compared to mRBC-CTRL and time points; 1-hour mRBC-CTRL vs mRBC-OVA-4-1BBL-IL-12 $P = 0.036$. **c**-**d**, Flow cytometry analyses of (**c**) CellTrace Far Red (CTFR) dye$^+$ mRBC signal associated with OT-1 cells (**c**) and % CD44$^+$ OT-1 cells (**d**). One-way ANOVA compared to mRBC-CTRL and time points; 17 hour % CTFR+ OT-1: mRBC-CTRL vs mRBC-OVA-4-1BBL-IL-12 $P = 0.0063$; 1 hour % CD44$^+$ OT-1 mRBC-CTRL vs mRBC-OVA-4-1BBL-IL-12 $P = 0.04$; 17 hour % CD44$^+$ OT-1 mRBC-CTRL vs mRBC-OVA-4-1BBL-IL-12 $P < 0.0001$; % CD44$^+$ OT-1 mRBC-OVA-4-1BBL-IL-12 1 hour vs 17 hour $P < 0.0001$. Data are depicted as mean ± s.d. and are representative of two independent experiments. Source data are provided as a Source Data file.

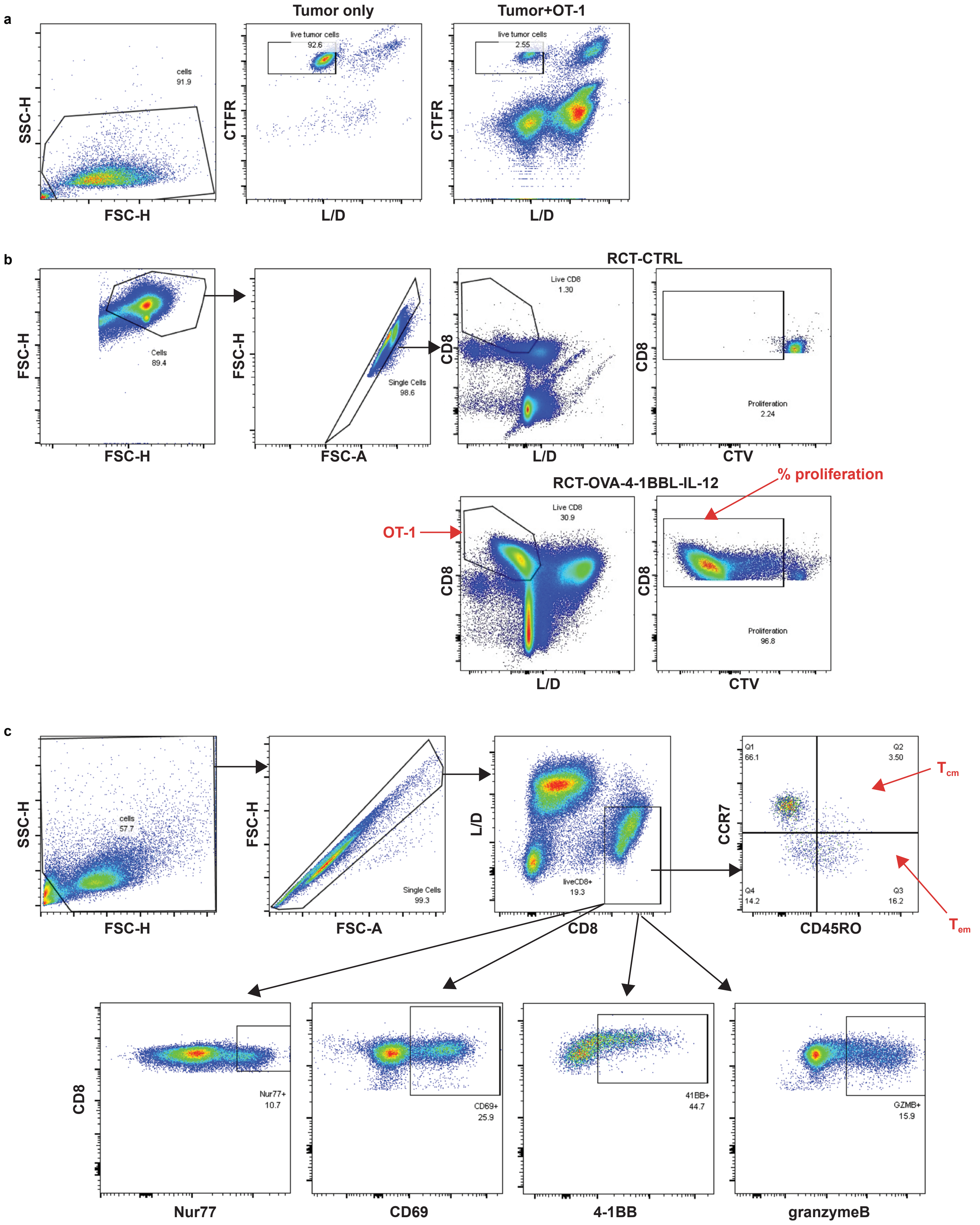

a
Tumor only
Tumor+OT-1
SSC-H
FSC-H
cells
91.9
CTFR
L/D
live tumor cells
92.6
live tumor cells
2.55
b
RCT-CTRL
FSC-H
Cells
89.4
FSC-A
Single Cells
98.6
CD8
L/D
Live CD8
1.30
CTV
Proliferation
2.24
RCT-OVA-4-1BBL-IL-12
% proliferation
OT-1
Live CD8
30.9
Proliferation
96.8
c
SSC-H
FSC-H
cells
57.7
FSC-A
Single Cells
99.3
L/D
CD8
liveCD8+
19.3
CCR7
CD45RO
Q1
66.1
Q2
3.50
Q4
14.2
Q3
16.2
Tcm
Tem
Nur77
Nur77+
10.7
CD69
CD69+
25.9
4-1BB
41BB+
44.7
granzymeB
GZMB+
15.9

Supplementary Fig. 7 Flow cytometry gating scheme for in vitro assays. a, Flow cytometry gating scheme for tumor killing assay in Fig. 1a and Supplementary Fig. 2a. **b**, Flow cytometry gating scheme for aAPC and OT-1 or pmel-1 co-culture assays in Supplementary Fig. 1c and 2b. **c**, Flow cytometry gating scheme for assays shown in Fig. 6. L/D, LIVE/DEAD™ stain; CTFR, CellTrace™ Far Red; CTV, CellTrace™ Violet; T_{em}, effector memory T cells; T_{cm}, central memory T cells.

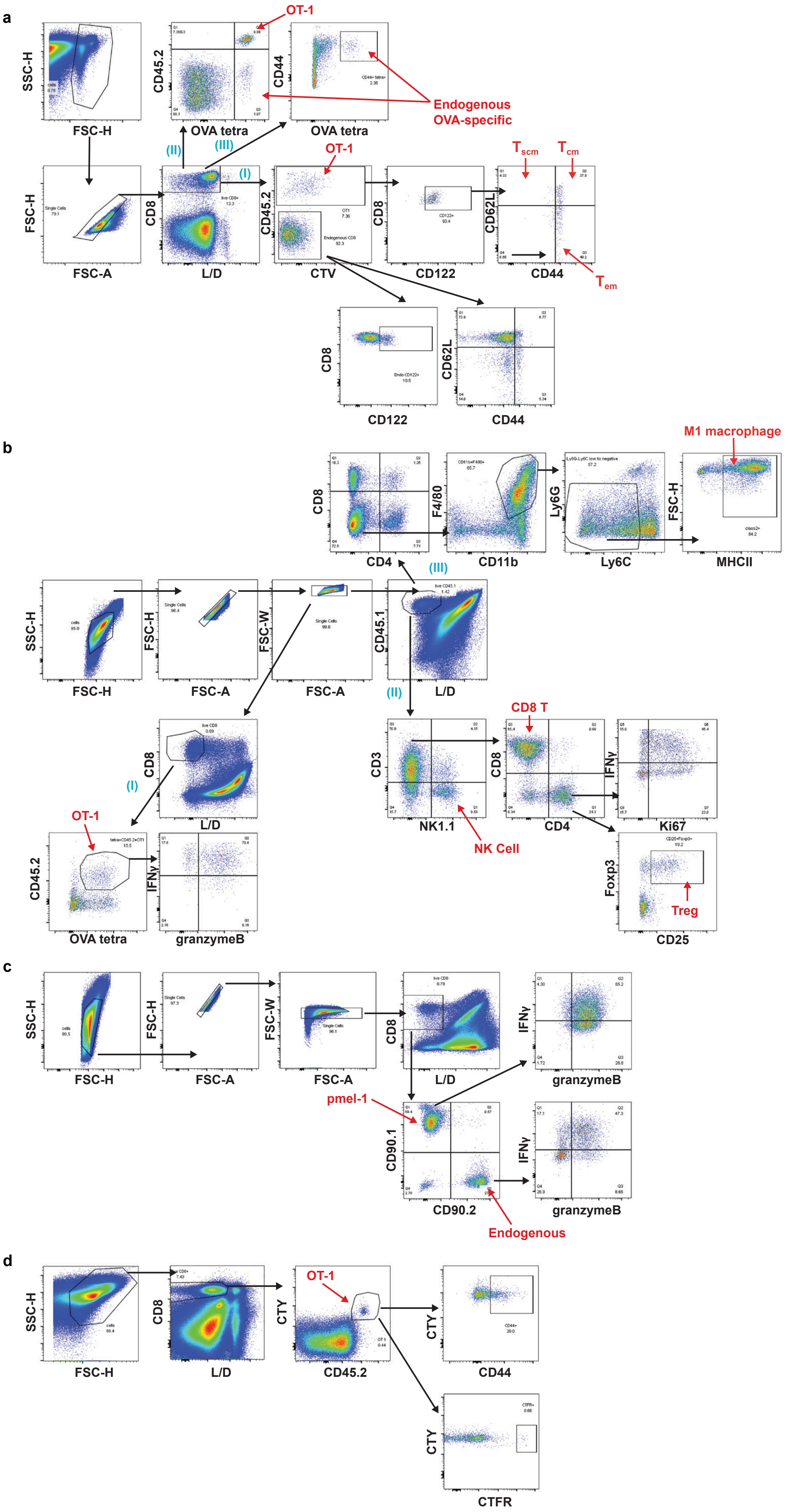

a
SSC-H
FSC-H
CD45.2
OVA tetra
OT-1
CD44
Endogenous
OVA-specific
(II)
(III)
(I)
FSC-A
CD8
L/D
CTV
CD122
CD62L
T_{scm}
T_{cm}
T_{em}
b
M1 macrophage
CD4
F4/80
CD11b
Ly6G
Ly6C
MHCII
FSC-W
CD45.1
CD3
NK1.1
CD8 T
NK Cell
IFNγ
Ki67
Foxp3
CD25
Treg
granzymeB
c
pmel-1
CD90.1
CD90.2
Endogenous
d
CTY
CTFR

Supplementary Fig. 8 Flow cytometry gating scheme for in vivo studies. a, Flow cytometry gating scheme for OT-1 T cell analyses in the blood in Fig. 1c, d, j, l (analysis I), Fig. 3d, e (analysis II), and Fig. 4c (analysis III). **b**, Flow cytometry gating scheme for OT-1 T cell analyses in the tumor in Fig. l, n (analysis I), Supplementary Fig. 4a-c (analysis II), and Supplementary Fig. 4d (analysis III). **c**, Flow cytometry gating scheme for pmel-1 T cell analyses in Fig. 5. **d**, Flow cytometry gating scheme for OT-1 T cell analyses in the spleen in Supplementary Fig. 6c, d. L/D, LIVE/DEAD™ stain; CTV, CellTrace™ Violet; CTFR, CellTrace™ Far Red; CTY, CellTrace™ Yellow; T_{em}, effector memory T cells; T_{cm}, central memory T cells; T_{scm}, stem memory T cells.
