# Supplementary material for: Engineered red blood cells as an off-the-shelf allogeneic anti-tumor therapeutic
Source: Nat Commun. 2021 May 11;12:2637. doi: 10.1038/s41467-021-22898-3 (PMC8113241; doi:10.1038/s41467-021-22898-3)
Supplement: Supplementary file 2 — Descriptions of Additional Supplementary Files [file 41467_2021_22898_MOESM2_ESM.pdf]

Descriptions of Additional Supplementary Files

**Supplementary Movie 1**
**Description:** Live cell confocal videos that captured activated OT-1 cells (green) landed onto a layer of immobilized CellTrace Far Red dye labeled mRBC-CTRL cells (red).

**Supplementary Movie 2**
**Description:** Live cell confocal videos that captured activated OT-1 cells (green) landed onto a layer of immobilized mRBC-OVA-4-1BBL-IL-12 that were conjugated with DL650-labeled 4-1BBL protein (red).
